# IL-6-mediated environmental conditioning of defective Th1 differentiation dampens antitumour immune responses in old age

**DOI:** 10.1038/ncomms7702

**Published:** 2015-04-07

**Authors:** Hirotake Tsukamoto, Satoru Senju, Keiko Matsumura, Susan L. Swain, Yasuharu Nishimura

**Affiliations:** 1 Department of Immunogenetics, Graduate School of Medical Sciences, Kumamoto University, Honjo 1-1-1, Chuo-ku, Kumamoto 860-8556, Japan; 2 Department of Pathology, University of Massachusetts Medical School, 335 Plantation Street, Worcester, Massachusetts 01655, USA

## Abstract

Decline in immune function and inflammation concomitantly develop with ageing. Here we focus on the impact of this inflammatory environment on T cells, and demonstrate that in contrast to successful tumour elimination in young mice, replenishment of tumour-specific CD4^+^ T cells fails to induce tumour regression in aged hosts. The impaired antitumour effect of CD4^+^ T cells with their defective Th1 differentiation in an aged environment is restored by interleukin (IL)-6 blockade or IL-6 deficiency. IL-6 blockade also restores the impaired ability of CD4^+^ T cells to promote CD8^+^ T-cell-dependent tumour elimination in aged mice, which requires IFN-γ. Furthermore, IL-6-stimulated production of IL-4/IL-21 through c-Maf induction is responsible for impaired Th1 differentiation. IL-6 also contributes to IL-10 production from CD4^+^ T cells in aged mice, causing attenuated responses of CD8^+^ T cells. These findings suggest that IL-6 serves as an extrinsic factor counteracting CD4^+^ T-cell-mediated immunity against tumour in old age.

## Abstract (web summary)

T-cell responses are dysregulated in aged humans and mice, which leads to poor antitumour responses. Here, the authors demonstrate that this phenomenon is at least partially due to an overproduction of IL-6 caused by ageing and its inhibitory effect on Th1 differentiation of tumour-specific CD4 T cells.

## Graphical abstract

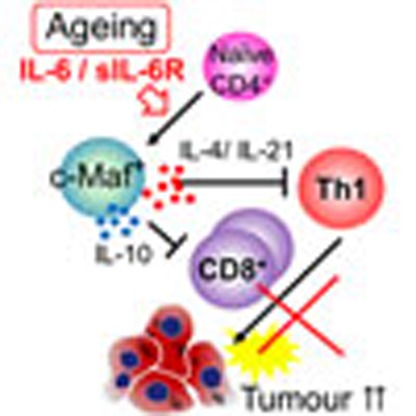

The emerging usefulness of tumour-specific T-cell-mediated cancer immunotherapies is increasingly appreciated. For a long time, antitumour responses of CD8^+^ T cells have been a main focus in the therapeutic effects. Currently, accumulating evidences have indicated that active immunotherapy inducing tumour-specific CD4^+^ T cells is also potentially powerful and broadly applicable for tumour rejection^1^,^2^,^3^,^4^. CD4^+^ T cells participate in tumour elimination by helping to activate other immune components such as CD8^+^ T cells, natural killer cells and macrophages^1^,^5^,^6^, exhibiting direct cytotoxicity against tumour cells^3^, and driving tumour cells into senescence^4^. An increase in interferon (IFN)-γ-producing T helper (Th)1 cells has been recognized as an antitumour immune signature in cancer patients^5^,^7^, because favourable prognosis is closely correlated with high expression of Th1-related genes, *Ifng* and *Tbx21* (T-bet)^5^. In contrast, Th2 rather than Th1 cells are predominantly increased in patients with advanced cancer^7^ and aged individuals^8^,^9^. Therefore, it has been assumed that strategies to promote the activation of tumour-specific Th1 cells would be useful for effective cancer immunotherapy.

Immune-based approaches are potentially less toxic than chemo- or radiotherapy. From this perspective, immunotherapy may be suitable for older cancer patients. However, immune responses become compromised during ageing. Age-related defects including both the relatively low number and the dysfunction of aged T cells, appear to not only increase cancer incidence in later life, but also to decrease the effectiveness of immunotherapy to mount T-cell responses against cancers, which leads to high morbidity and mortality in the elderly population^10^. Our and other studies have demonstrated that the functions of CD4^+^ T cells are profoundly altered by the ageing process^11^,^12^,^13^. The lower efficacy of CD4^+^ T-cell-mediated immune responses in old age can be attributable to several mechanisms including T-cell-intrinsic^11^,^12^,^13^ and -extrinsic effects^14^. However, the influences of age-related changes in CD4^+^ T-cell-mediated immune responses on the effectiveness of cancer immunotherapy are obscure because much of our understanding about antitumour immunotherapy is based on studies with young animals. To design effective immunotherapeutic interventions specifically tailored to older cancer patients, it is important to know why T-cell functions are diminished in old age, and how to potentiate the aged immune system.

It has been assumed that the chronic low-grade inflammation that accompanies ageing plays a role in the pathogenesis of several age-associated diseases including cancer^10^,^15^,^16^,^17^. For instance, increased levels of the pro-inflammatory cytokine interleukin (IL)-6 are correlated with frailty in these patients^15^,^18^. In addition, various studies have revealed that IL-6 is one of the adverse prognostic factors for cancer progression and has tumour-promoting effects^19^. However, little attention has been paid to an influence of excessive levels of IL-6 on T-cell-mediated antitumour responses in old age.

In the present study, we asked whether CD4^+^ T-cell dysfunction in aged hosts could be reversed by complementation with young tumour-specific CD4^+^ T cells. However, young tumour-specific CD4^+^ T cells primed in aged mice did not mount protective immune responses against tumour. Thus, we focused on an altered cytokine milieu in aged animals, and evaluated the influence of IL-6, which found to be abundantly present in aged mice and humans, on the poor CD4^+^ T-cell-mediated antitumour responses. Although IL-6 did not diminish or promote *in vivo* expansion of CD4^+^ T cells in response to vaccination, the age-associated increase in IL-6 dampened Th1 differentiation of CD4^+^ T cells and subsequent induction of tumour-specific CD8^+^ T cells, and thereby promoted cancer progression in aged mice. Our findings also suggest that IL-6-induced c-Maf/IL-4/IL-21/IL-10 axis is a mechanistic feature of the aged environmental conditioning of CD4^+^ T cells.

## Results

### CD4^+^ T-cell-mediated therapy is less effective in aged mice

**Figure 1:**
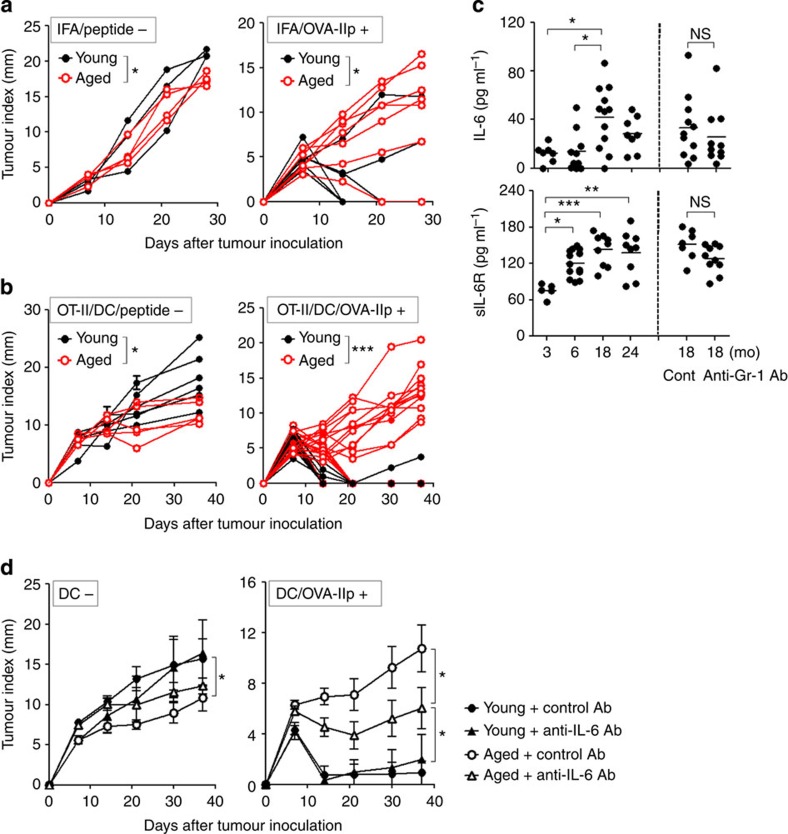
Tumour-specific CD4^+^ T-cell-mediated protective antitumour immunity is impaired with age-dependent increases of IL-6 and sIL-6R. (**a**) Young and aged mice were vaccinated with or without OVA-IIp emulsified in IFA. Seven days after immunization, mice were inoculated with MCA-OVA. (**b**) CD45.1^+^ young naive OT-II T cells were transferred into young or aged CD45.2^+^ mice. The mice were then immunized by transfer of DCs pulsed with or without OVA-IIp. Tumour inoculation was then performed. Each line represents the kinetics of tumour growth in an individual mouse (*n*=4–12 mice per group). *P<0.05, ***P<0.001, unpaired Student's *t*-test. (**c**) Quantitation of IL-6 (upper) and sIL-6R (lower) in sera from 3-, 6-, 18- or 24-month-old mice. Control or anti-Gr-1 Ab was injected 5 days before serum harvest. Each point represents an individual mouse. (**d**) OT-II cell transfer and immunization were performed as described in **b**. Mice were treated with control or anti-IL-6 Ab on day −1 and day 1. Six days after immunization, MCA-OVA were injected. Representative data from three independent experiments with similar results are shown (*n*=6–8 mice per group). *P<0.05, **P<0.01, ***P<0.001, analysis of variance followed by Tukey's *post hoc* test. NS, not significant.

We examined the effect of CD4^+^ T-cell-mediated antitumour vaccination using MCA205 tumour cells expressing ovalbumin (OVA) as a surrogate antigen (hereafter referred to as MCA-OVA). As previously reported in cancer patients^10^, tumour masses grew more slowly in aged mice than in young mice (Fig. 1a, left). In young mice, tumour outgrowth was prevented by vaccination with OVA peptide recognized by major histocompatibility complex (MHC) class-II-restricted CD4^+^ T cells (referred to as OVA-IIp). In contrast, vaccinated aged mice did not eliminate the tumours (Fig. 1a, right). This might be due to the reduced number of aged CD4^+^ T cells and their intrinsic defect in ability to expand in response to antigenic stimulation^11^,^12^,^13^.

Therefore we asked whether transfer of young OT-II cells and immunization with OVA-IIp-pulsed young dendritic cells (DCs) overcome these defects in aged mice. When MCA-OVA were inoculated, OT-II cell transfer was strikingly effective for tumour rejection in young hosts, but had little therapeutic effect in aged mice (Fig. 1b). These results suggest that the antitumour immune responses initiated by tumour-specific CD4^+^ T cells are dampened in aged mice, and that the age-associated dysfunction in CD4^+^ T cells *in vivo* is partly due to the extrinsic factors surrounding CD4^+^ T cells in aged hosts.

### IL-6 is suppressive for CD4-mediated antitumour immunity

As previously reported in human^18^, serum IL-6 levels were elevated with increasing age (Fig. 1c). As well as IL-6, the levels of soluble IL-6 receptor (sIL-6R), which forms a complex with IL-6 and promotes to transduce intracellular signal through glycoprotein 130 (ref. 20), were also systemically increased with ageing. To determine whether circulating IL-6 influences the ability of CD4^+^ T cells to control tumours, we employed IL-6 blockade combined with OT-II cell transfer. Following tumour challenge, the growth of MCA-OVA was slightly faster in young mice than in aged mice receiving control antibody (Ab) singly (Fig. 1d, left). Young hosts exhibited marked tumour eradication regardless of IL-6 blockade when donor CD4^+^ T cells were primed with peptide-pulsed DCs, whereas as expected the antitumour effect of CD4^+^ T cells was impaired in control Ab-treated aged mice. On the other hand, the combination of vaccination with anti-IL-6 Ab treatment significantly improved the antitumour effect of OT-II cells in aged mice (Fig. 1d, right).

**Figure 2:**
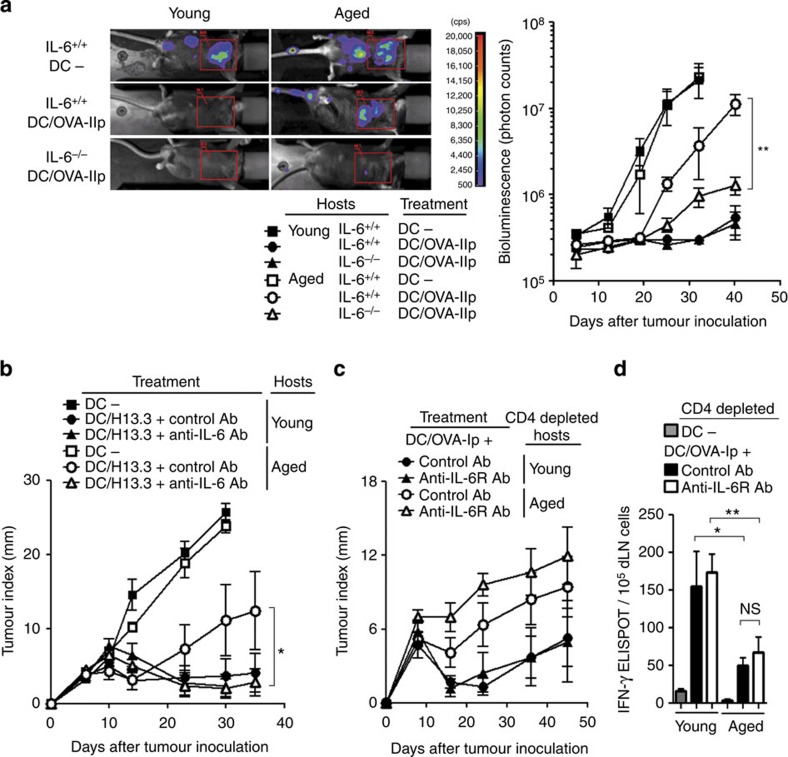
IL-6 increased in the aged environment is responsible for the impairment of CD4^+^ T-cell-mediated antitumour immunity. (**a**) OT-II cells were transferred into young or aged IL-6^+/+^ or IL-6^−/−^ mice. Immunization was performed as in Fig. 1b. Five days after immunization, luciferase-expressing MO4 were intravenously injected for the pulmonary metastatic model. Luminescence images at day 32 (left) and kinetics of photon counts per mouse (right) are shown. (**b**) Young and aged mice were immunized by the transfer of EnvH13.3 peptide-pulsed DCs, and were treated with control Ab or anti-IL-6R Ab 1 day before and after immunization. On day 6 post immunization, RMA tumour cells were inoculated and their growth was monitored. (**c**,**d**) Young and aged mice were treated with anti-CD4 Ab. After 2 days, the mice were immunized with DCs pulsed with OVA-Ip (SIINFEKL), and then inoculated with MCA-OVA. Tumour progression was monitored with time (**c**). Five days after tumour inoculation, draining LNs were harvested and re-stimulated with OVA-Ip-pulsed DC *ex vivo*. IFN-γ production was determined by the ELISPOT assay (**d**). Representative from two or three experiments are shown as the mean±s.e.m. (*n*=5–10 mice per group). *P<0.05, **P<0.01, analysis of variance followed by Tukey's *post hoc* test. NS, not significant.

The negative impact of the aged environment on CD4^+^ T-cell responses might be manifested more prominently in the case of malignant cells with vigorous progression and metastasis. Therefore, employing a metastatic model of aggressive melanoma, we re-evaluated the CD4^+^ T-cell-mediated antitumour effects in aged mice. As was observed with the MCA-OVA, OT-II cells primed with peptide-pulsed DCs were ineffective for metastatic melanoma in aged IL-6^+/+^ mice compared with those in young IL-6^+/+^ or IL-6^−/−^ mice (Fig. 2a). However, when immunized in aged IL-6^−/−^ mice, their efficacy was significantly improved, suggesting that increased IL-6 levels in aged hosts dampened the function of tumour-specific CD4^+^ T cells.

We also investigated the suppressive effect of IL-6 on more physiological immune responses of endogenous CD4^+^ T cells in protecting aged mice against the outgrowth of murine leukaemia virus (MuLV)-induced lymphoma, RMA^21^. When mice were immunized with DCs pulsed with EnvH13. 3 peptide, which was a natural viral epitope derived from MuLV and was recognized by I-A^b^-restricted CD4^+^ T cells, the vaccination delayed tumour outgrowth in young mice in comparison with that in non-vaccinated young mice^21^ or in vaccinated aged mice (Fig. 2b). Moreover, in aged mice treated with DC vaccination together with anti-IL-6 Ab, progression of RMA tumour was significantly repressed as observed in the MCA-OVA model.

Even when endogenous CD4^+^ T cells were depleted and OT-II cells was not transferred, immunization with DCs pulsed with SIINFEKL (OVA-Ip), which is recognized by MHC class-I-restricted OVA-specific CD8^+^ T cells, induced moderate antitumour responses in young mice (Fig. 2c). However, in aged mice, immunization with DCs that primed the endogenous OVA-specific CD8^+^ T cells without OT-II transfer was less effective than that observed in young mice. Moreover, IL-6 blockade neither improved nor diminished the antitumour immunity in CD4^+^ T-cell-depleted and CD8^+^ T-cell-primed aged mice. These antitumour effects were closely correlated with IFN-γ responses of OVA-Ip (tumour)-specific CD8^+^ T cells, which was not affected by anti-IL-6 Ab administration (Fig. 2d), although aged CD8^+^ T-cell responses were decreased as compared with young ones because of their quantitative decline in CD8^+^ T cells^13^. These results ruled out the possibility that the beneficial effect of IL-6 blockade was exerted directly through CD8^+^ T-cell-mediated antitumour immunity. Therefore, heightened IL-6 appeared to act predominantly on tumour-specific CD4^+^ T cells in aged mice.

### The aged environment dampens CD4^+^ T-cell proliferation

**Figure 3:**
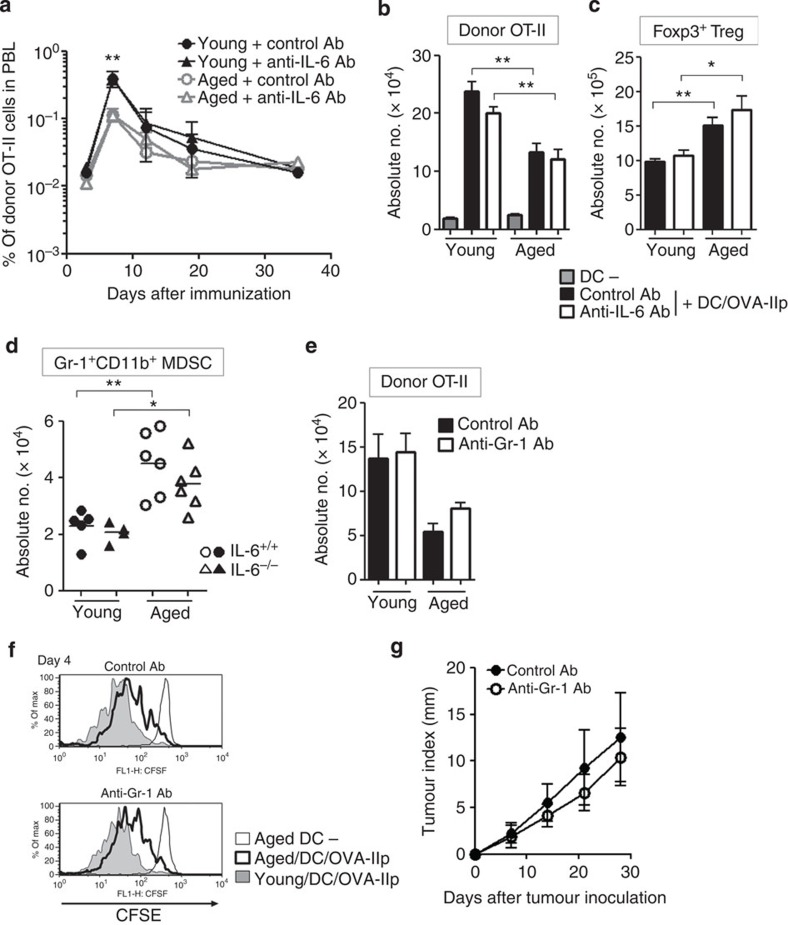
The aged environment dampens antigen-induced proliferation of CD4^+^ T cells. (**a**–**c**) OT-II cell transfer, immunization and Ab treatment were performed as described in Fig. 1d. The frequency of donor CD45.1^+^OT-II cells in PBL was monitored over time (**a**), and the total number of donor OT-II cells (**b**) and CD4^+^Foxp3^+^Treg cells (**c**) in spleen and LNs were determined 6 days after immunization. (**d**) The number of Gr-1^+^CD11b^+^ MDSC in spleen and LN cells from young or aged IL-6^+/+^ or IL-6^−/−^ mice were determined. (**e**–**g**) Anti-Gr-1 Ab was injected 5 days before transfer of the CFSE-labelled OT-II cells, followed by immunization. The total number at day 6 (**e**) and CFSE profile at day 4 (**f**) of donor OT-II cells under the indicated conditions are shown. Seven days after immunization, MCA-OVA were inoculated into the aged mice. The outgrowth in aged mice was measured over time (**g**). The values are mean±s.e.m. (*n*=6 per group); *P<0.05, **P<0.01, analysis of variance followed by Tukey's *post hoc* test. Representative data from two or three experiments with similar results are presented.

It was possible that in the aged environment, CD4^+^ T cells failed to expand efficiently in response to antigenic stimulation, resulting in poor antitumour immunity. To test this possibility, we examined the proportion of young donor OT-II cells in aged mice after immunization. Vigorous increases of donor OT-II cells were observed in peripheral blood within 7 days in both control and anti-IL-6 Ab-treated young hosts, and the donor cells contracted afterwards (Fig. 3a). In contrast, donor T-cell expansion was less prominent in aged hosts than in young counterparts in the blood, and in spleen and lymph nodes (LNs) when vaccinated with DCs (Fig. 3a,b), or when the homeostatic division was induced in irradiated mice (Supplementary Fig. 1). Moreover, anti-IL-6 Ab treatment did not improve the inadequate expansion of donor OT-II cells in aged mice.

CD4^+^Foxp3^+^ regulatory T cells (Treg) could potentially act as an extrinsic factor that affects the T-cell responses in aged hosts, because they were abundantly present in aged humans^22^ and mice (Fig. 3c)^23^. However, control and anti-IL-6 Ab-treated aged mice had similar number of Treg. Another T-cell-suppressive component was Gr-1^+^CD11b^+^ myeloid-derived suppressor cells (MDSCs), which were also expanded during ageing (Supplementary Fig. 2)^23^. Interestingly, IL-6 deficiency did not alter the age-related increase in MDSC proportion (Fig. 3d). To test their suppressive effect on antigen-induced expansion of CD4^+^ T cells, we depleted Gr-1^+^ cells including MDSC using anti-Gr-1 Ab. Although the total number and carboxyfluorescein diacetate succinimidyl ester (CFSE) profile of donor OT-II cells revealed their reduced proliferation in aged mice, no measurable changes in their expansion were observed between anti-Gr-1 Ab-treated and control aged hosts (Fig. 3e,f). Furthermore, unlike anti-IL-6 Ab, anti-Gr-1 Ab treatment had no effects on CD4^+^ T-cell-mediated antitumour immunity in aged mice (Fig. 3g). These results suggest that the suppressive effects of accumulated Treg, MDSC or other Gr-1^+^ cells such as neutrophils were independent of the improved efficacy of CD4^+^ T-cell-mediated antitumour immunity in IL-6-neutralized aged mice.

### IL-6 hampers Th1 development in an aged environment

**Figure 4:**
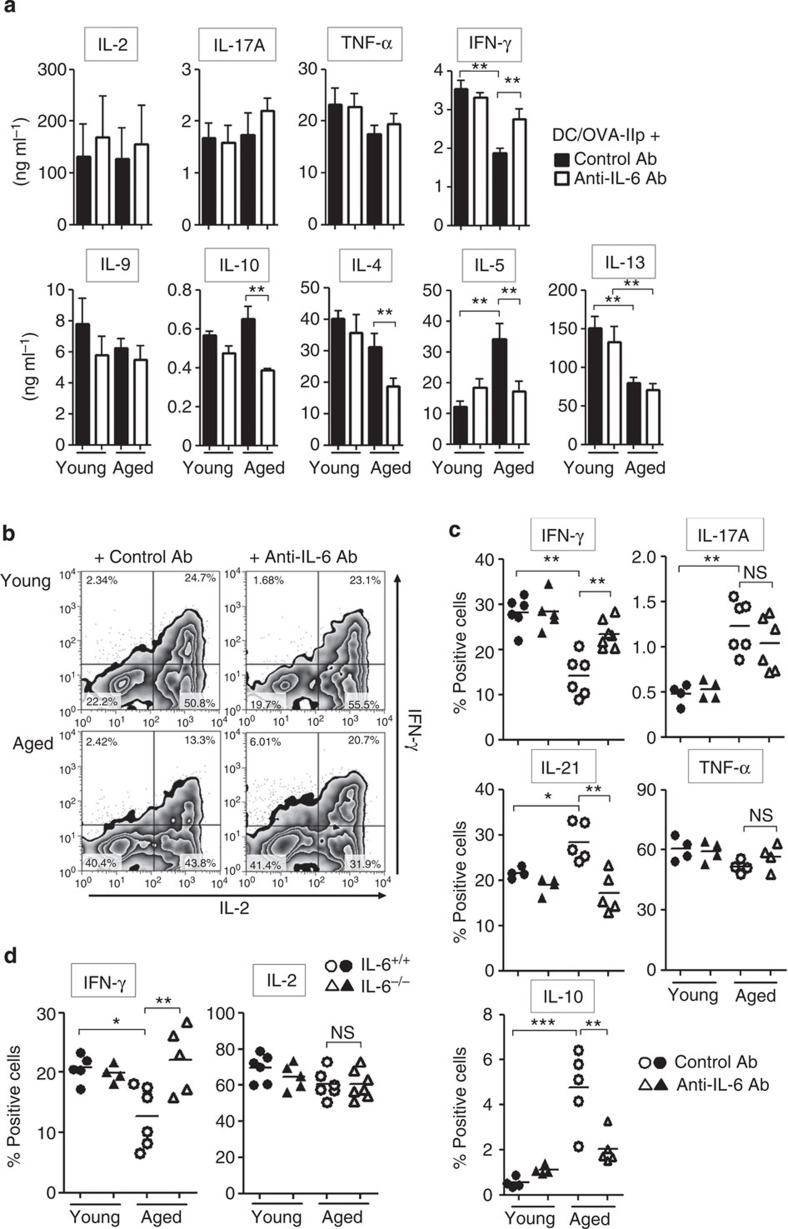
Age-related increase in IL-6 attenuates Th1 differentiation of antigen-primed CD4^+^ T cells. (**a**) OT-II cell transfer, immunization and Ab treatment were performed as described in Fig. 1d. Six days after immunization, CD4^+^CD62L^lo^ cells were sorted from the spleen and LNs of young and aged mice (details are described in Methods), and then were re-stimulated *ex vivo* with OVA-IIp-pulsed DCs for 36 h. The culture supernatant was harvested, and the concentrations of the indicated cytokines were measured. The values are mean±s.e.m. (*n*=4 per group). (**b**–**d**) OT-II cell transfer, immunization and anti-IL-6 Ab injection were performed. IL-6^+/+^ or IL-6^−/−^ mice were utilized as hosts in **d**. Donor OT-II cells were isolated 6 days after immunization, and re-stimulated with phorbol-12-myristate-13-acetate/ionomycin. Cytokine production was assessed by intracellular staining. Representative dot plots (**b**) and individual values along with the mean (**c**,**d**) in the indicated cytokine-positive fractions are shown. Representative data from 3–4 independent experiments with similar results are shown. *P<0.05, **P<0.01, ***P<0.001, analysis of variance followed by Tukey's *post hoc* test. NS, not significant.

It was hypothesized that IL-6 upregulated in aged mice altered the cytokine production by tumour-specific CD4^+^ T cells to prevent them from attacking the tumours. Indeed, when CD62L^lo^ effector CD4^+^ T cells including OT-II cells primed *in vivo* with or without IL-6 blockade were re-stimulated with OVA-IIp *ex vivo*, we found that the levels of IFN-γ, IL-4, IL-5, IL-13 and IL-10, but not IL-2, IL-17A, tumour necrosis factor (TNF)-α or IL-9 produced from effector CD4^+^ T cells, were remarkably affected by the aged environment and IL-6 blockade (Fig. 4a). To test whether these changes were due to altered differentiation of effector CD4^+^ T cells *in vivo*, we assessed the ability of donor OT-II cells to produce cytokines on a per-cell basis. The frequency of IFN-γ-producing OT-II cells from aged hosts was significantly lower than that from young hosts (Fig. 4b,c). Of note, this defective Th1 differentiation of donor OT-II cells was significantly restored by IL-6 blockade in aged hosts. We found no difference between control and anti-IL-6 Ab-treated aged mice in their ability to produce IL-2, TNF-α and IL-17A (Fig. 4b,c). Conversely, the proportions of IL-21- and IL-10-producing cells were increased in aged mice, and these were diminished by IL-6 blockade (Fig. 4c; Supplementary Fig. 3a). Similar trends were also observed in messenger RNA (mRNA) expression of these cytokines in primed CD4^+^ T cells (Supplementary Fig. 3b). Furthermore, evaluation of cytokine production from donor OT-II cells primed in IL-6-deficient aged mice confirmed that the IL-6-dependent impairment of Th1 differentiation in aged hosts (Fig. 4d). In line with no significant effect of anti-Gr-1 Ab on IL-6 production (Fig. 2a) and antitumour immune response in aged mice (Fig. 3g), depletion of Gr-1^+^ cells including MDSC did not change the cytokine profiles in donor OT-II cells primed in aged mice (Supplementary Fig. 2b). These results suggest that the increased levels of IL-6 serve as a major mechanism underlying the impaired Th1 differentiation of donor CD4^+^ T cells in aged mice. The impaired Th1 response in donor OT-II cells in aged mice was consistent with their reduced expression of IFN-γ-inducible chemokine receptor, CXCR3 (ref. 24), which was upregulated by IL-6 blockade (Supplementary Fig. 2c,d). Although CD25 expression was also decreased in donor cells primed in aged mice as compared with those in young mice, IL-6 blockade did not alter its expression as with other surface markers. Increased expression of the markers for exhausted T cells such as PD-1 were not found in donor OT-II cells in aged mice (Supplementary Fig. 2d).

**Figure 5:**
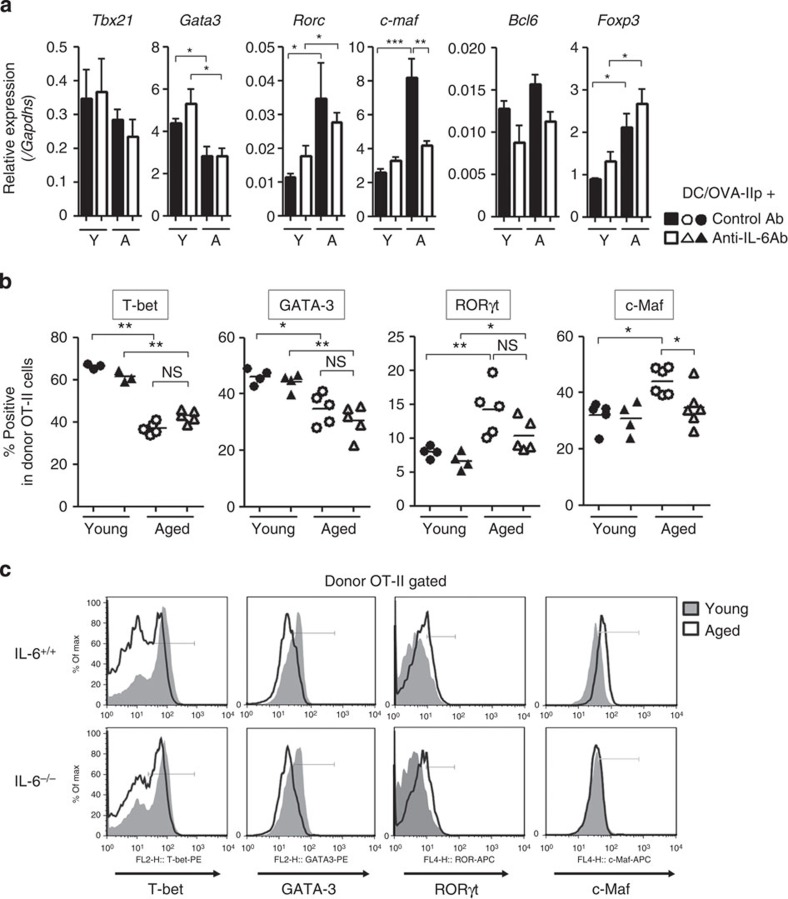
IL-6 and the aged environment alter the expression profile of transcription factors in CD4^+^ T cells. OT-II cell transfer, immunization and anti-IL-6 Ab injection were performed as in Fig. 1d. (**a**) After 5 days, total CD4^+^ T cells were sorted from spleen and LNs, and RNA was isolated from them. mRNA expression of indicated transcription factors was analysed by real-time quantitative PCR. Shown is relative value to *Gapdh* expression (mean±s.e.m. with *n*=4–6 per group). Y, young hosts; A, aged hosts. (**b**,**c**) Six days after immunization, donor OT-II cells were analysed for protein expression of indicated transcription factors. Individual values along with the mean from Ab-treated IL-6^+/+^ mice (**b**) and the representative histograms from IL-6^+/+^ or IL-6^−/−^ mice (**c**) are shown. Representative from three independent experiments are shown. *P<0.05, **P<0.01, ***P<0.001, analysis of variance followed by Tukey's *post hoc* test. NS, not significant.

We also examined the effect of age-related increase of IL-6 on gene expression of key transcription factors that regulate T-cell differentiation. In total CD4^+^ T cells isolated from OT-II-transferred and vaccinated mice, the expression levels of *Tbx21*, which encodes T-bet and regulates Th1 differentiation^25^, tended to be decreased in aged mice as compared with in young mice, but the difference was not significant. Moreover, *Gata3* expression that regulates Th2 differentiation was also lower in CD4^+^ T cells from vaccinated aged mice than that in young mice. In contrast, *Foxp3* and Th17-specific transcription factor *Rorc* were upregulated, but those expressions were not altered by IL-6 blockade in either young or aged mice (Fig. 5a). As was observed with *Rorc* expression, *c-maf* expression in CD4^+^ T cells was markedly augmented in aged hosts, but was repressed by IL-6 blockade. To analyse the expression of transcription factors in antigen-specific CD4^+^ T cells more precisely, their protein expressions in donor OT-II cells were examined at the single-cell level. As expected, similar patterns were observed in protein expression of donor OT-II cells primed in young or aged mice (Fig. 5b). Of note, only c-Maf expression was altered by both host age and IL-6 level, and this was confirmed in donor OT-II cells primed in IL-6-replete and -deficient aged mice (Fig. 5c).

### Defective IFN-γ response in IL-6-mediated CD4^+^ T-cell dysfunction

**Figure 6:**
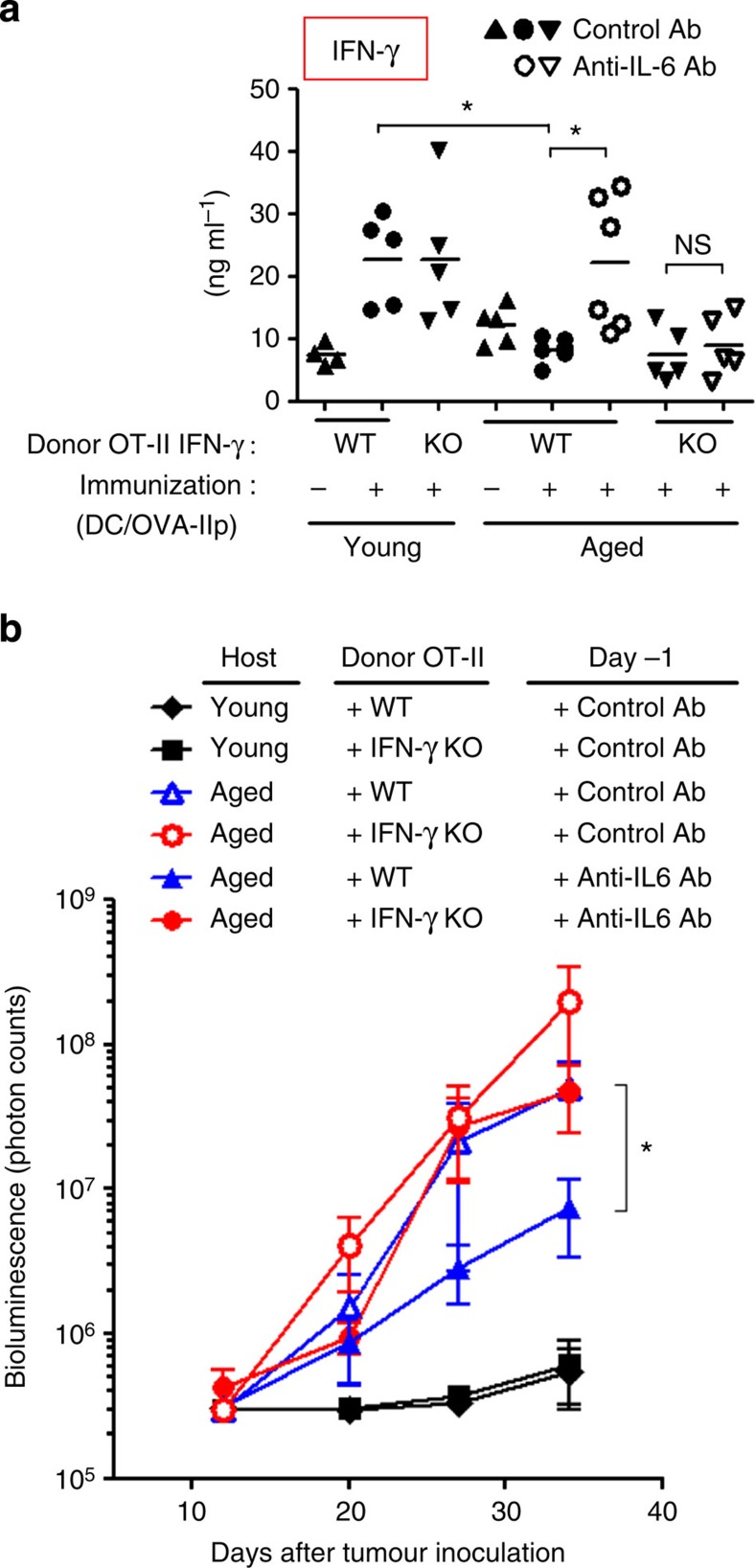
Tumour-specific CD4^+^ T-cell-derived IFN-γ is a prerequisite for the effect of IL-6 blockade on antitumour activity in aged mice. OT-II cells from young wild-type (WT) or IFN-γ-deficient (IFN-γ knockout (KO)) mice were transferred into young and aged mice. Immunization and tumour inoculation were performed as described in Fig. 2a. (**a**) One week after tumour inoculation, IFN-γ concentration in serum was determined by an enzyme-linked immunosorbent assay. Individual values along with the mean are shown. (**b**) The *in vivo* growth of luciferase-expressing MO4 cells was monitored over time, and is shown as the photon counts in luminescence images. Data shown are representatives from two experiments with similar results (mean±s.e.m. with *n*=4–6 per group; *P<0.05, analysis of variance followed by Tukey's *post hoc* test). NS, not significant.

We found that the serum level of IFN-γ was augmented in OT-II-transferred and vaccinated young mice in response to tumour inoculation (Fig. 6a). Furthermore, the relatively lower levels of IFN-γ in vaccinated aged mice were improved by the treatment with anti-IL-6 Ab, which was consistent with more Th1 differentiation in donor OT-II cells. However, aged mice that were transferred with IFN-γ-deficient OT-II cells did not evoke IFN-γ induction even when anti-IL-6 Ab was administered.

Given that production of IFN-γ, one of the most critical cytokines for antitumour immunity^3^,^5^,^7^, was defective in tumour-specific CD4^+^ T cells in aged mice, we next examined whether IL-6-induced impairment of Th1 differentiation indeed contributed to the age-associated dysfunction in antitumour responses of CD4^+^ T cells. As shown in Fig. 6b, vigorous progression of pulmonary metastatic tumour was prevented in young mice when either IFN-γ-sufficient or -deficient CD4^+^ T cells were transferred, whereas vaccinated aged mice failed to suppress the tumour outgrowth. However, IL-6 blockade significantly improved the therapeutic efficacy of wild-type (WT) donor OT-II cells in aged mice, as demonstrated before. In contrast, this improvement was not observed when aged mice received combined treatment with anti-IL-6 Ab and IFN-γ-deficient T cells. These results suggest that IL-6 blockade improves the antitumour effect of CD4^+^ T cells through augmented IFN-γ production in aged mice.

### Decreased CD8 help by CD4^+^ T cells in an aged environment

**Figure 7:**
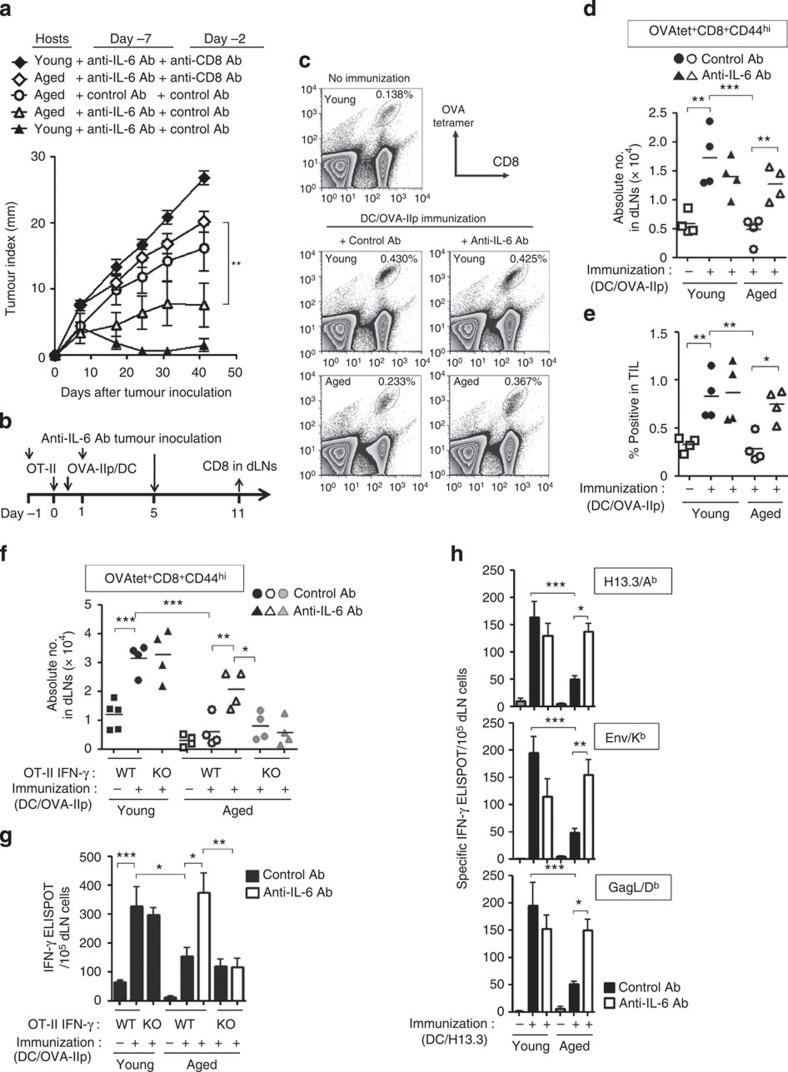
Th1-dependent helper activity for tumour-specific CD8^+^ T cells is diminished by IL-6 in aged mice. (**a**) T-cell transfer, immunization and Ab treatment were performed as in Fig. 1d. Five days after immunization, the mice were injected with anti-CD8 or control Ab. MCA-OVA was inoculated on day 0. Data for tumour growth are mean±s.e.m. (*n*=8–10 mice per group). (**b**–**e**) MCA-OVA were inoculated 5 days after OT-II transfer and immunization. Another 6 days later, OVA-specific CD8^+^ T cells in tumour-draining LNs (c and d) and tumour-infiltrated lymphocytes (TILs) (**e**) were analysed. Representative plots (**c**) and the total number (**d**) of OVA-Ip/H-2K^b^-tetramer^+^CD8^+^CD44^hi^ cells are shown. (**f**,**g**) WT or IFN-γ-deficient (knockout (KO)) OT-II cells were transferred into young and aged mice. OVA-specific CD8^+^ T cells in young or aged hosts that were treated as described in **b** were determined by OVA-Ip/H-2K^b^-tetramer staining (**f**). OVA-Ip-specific CD8^+^ T-cell response in tumour-draining LNs was also assessed by IFN-g ELISPOT (**g**). (**h**) Immunization with EnvH13.3-pulsed DC and RMA inoculation in young or aged mice were performed as described in Fig. 2b. Five days after tumour inoculation, indicated tumour-associated peptide-specific IFN-γ production in tumour-draining LNs were assessed by ELISPOT. Representative data from 2–3 experiments are shown (mean±s.e.m. with *n*=4–5 per group). *P<0.05, **P<0.01, ***P<0.001 analysis of variance followed by Tukey's *post hoc* test.

MCA-OVA neither express MHC-II nor present OVA peptide to CD4^+^ T cells^26^. The antitumour effects of CD4^+^ T cells might be exerted through other host-derived components such as tumour-specific CD8^+^ T cells. Therefore, we investigated whether aged hosts depleted of CD8^+^ T cells could mount antitumour immunity when CD4^+^ T-cell therapy was combined with IL-6 blockade. As demonstrated before, even in aged mice tumour outgrowth was substantially diminished by donor OT-II cells primed concomitantly with IL-6 blockade when endogenous CD8^+^ T cells were retained (Fig. 7a). However, the depletion of CD8^+^ T cells abrogated this antitumour effect, suggesting that therapeutic effect of tumour-specific CD4^+^ T cells in IL-6-neutralized aged mice was mediated through CD8^+^ T cells.

Neutralization of IL-6 activity influenced only at priming of donor CD4^+^ T cells with DCs, but did not affect CD8^+^ T-cell activation, because the DCs used in this vaccination were only pulsed with OVA-IIp, and tumour-specific CD8^+^ T cells should be exposed to antigen only after the challenge with MCA-OVA (Fig. 7b). We thus analysed the ability of effector CD4^+^ T cells primed in aged hosts to promote tumour-specific CD8^+^ T-cell responses after tumour challenge. Six days after tumour challenge, young mice in which donor OT-II cells had been primed, induced OVA-specific CD8^+^ T cells in tumour-draining LNs and in tumour more efficiently than mice possessing naive OT-II cells without DC vaccination, regardless of anti-IL-6 Ab treatment (Fig. 7c–e; Supplementary Fig. 4). In contrast, fewer OVA-specific CD8^+^ T cells were promoted by donor OT-II cells primed in aged mice. Notably, their helper activity for OVA-specific CD8^+^ T cells was markedly improved in aged mice where OT-II cells were primed in the presence of anti-IL-6 Ab.

In the case of RMA tumour in aged mice, IFN-γ response of tumour (MuLV EnvH13.3)-specific CD4^+^ T cells was restored by IL-6 blockade (Fig. 7f). On the other hand, EnvH13.3-specific IL-4 and IL-10 production were reciprocally downregulated by IL-6 blockade in aged mice (Supplementary Fig. 5). Furthermore, vaccination that primed the tumour-specific CD4^+^ T cells enhanced the responses of tumour (Env/K^b^ and GagL/D^b^)^21^-specific CD8^+^ T cells in aged mice only when anti-IL-6 Ab was administered at the CD4^+^ T-cell-priming phase (Fig. 7f).

We also examined the requirement of donor CD4^+^ T-cell-derived IFN-γ for promoting the response of tumour-specific CD8^+^ T cells. In agreement with the results of tumour growth (Fig. 6), no remarkable induction of OVA-specific CD8^+^ T cells and their functional enhancement that was monitored by IFN-γ response were observed in aged mice transferred with IFN-γ-deficient OT-II cells, regardless of IL-6 blockade (Fig. 7g,h). These results suggest that CD4^+^ T-cell-derived IFN-γ contribute to promoting the tumour-specific CD8^+^ T-cell response in anti-IL-6 Ab-treated aged mice.

### IL-6 dampens Th1 development through a c-Maf/IL-4/IL-21 axis

**Figure 8:**
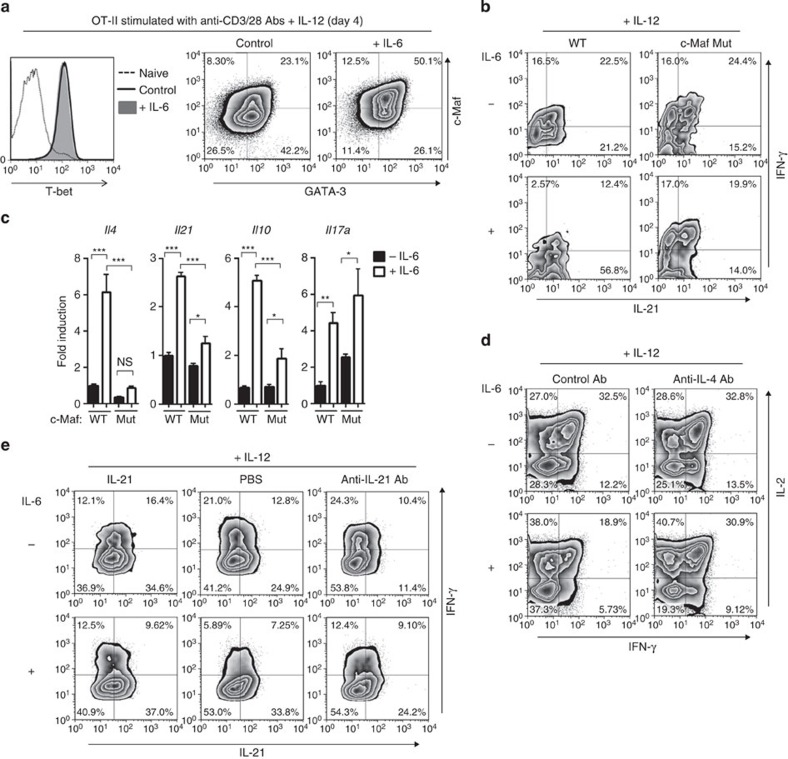
IL-6-induced IL-4/IL-21 production is responsible for the defect of Th1 differentiation. Young naive CD4^+^ T cells were stimulated with anti-CD3 and anti-CD28 Abs plus exogenous IL-12 *in vitro*. (**a**) Four days after stimulation, expressions of the indicated transcription factors were analysed. (**b**,**c**) Naive polyclonal CD4^+^ T cells from homozygous c-Maf-mutant mice (c-Maf Mut) or littermate control mice (WT) were stimulated in the presence or absence of IL-6. Five days after stimulation, effector cells were re-stimulated with phorbol-12-myristate-13-acetate/ionomycin. Representative plots of cytokine-producing cells are shown (**b**). Indicated cytokine mRNA expression at day 3 was also assessed by real-time quantitative PCR (**c**). Results are shown as mean±s.e.m. with *n*=4–6 per group; *P<0.05, **P<0.01, ***P<0.001, analysis of variance followed by Tukey's *post hoc* test. NS, not significant. (**d**,**e**) Naive OT-II cells were stimulated in the presence of indicated cytokines or Abs. Five days after stimulation, effector cells were re-stimulated and analysed for the ability to produce IFN-γ, IL-2 and/or IL-21. Representative plots are shown. The data are representative of at least three independent experiments. NS, not significant; PBS, phosphate-buffered saline.

**Figure 9:**
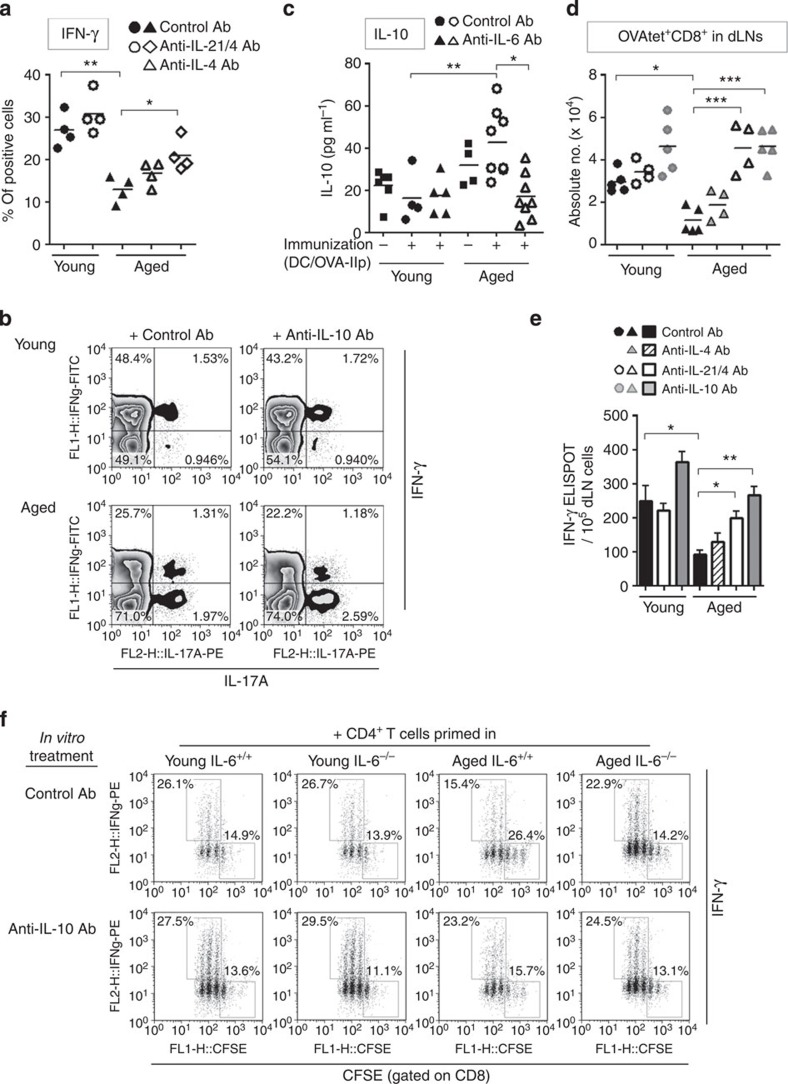
IL-6-induced IL-4/IL-21/IL-10 production is responsible for the defective CD8 help in aged mice. (**a**,**b**) OT-II cells were primed in young or aged mice at day 0 as in Fig. 1b, and then were treated with control Ab, anti-IL-4 and anti-IL-21 Abs (**a**) or anti-IL-10 Ab (**b**) on days 3 and 4. Six days after immunization, the frequencies of IFN-γ^+^ cells in the donor OT-II cells were determined. (**b**) Representative plots. (**c**) OT-II transfer, immunization, Ab treatment and MCA-OVA inoculation were performed as in Fig. 1d. Four days after tumour inoculation, IL-10 concentration in serum was determined. (**d**,**e**) OT-II transfer, immunization and treatments of anti-IL-4 and anti-IL-21 Abs were performed as in **a**. Five days after immunization, mice were inoculated with MCA-OVA. Anti-IL-10 Ab was injected twice at 2 and 3 days after tumour inoculation. Five days after tumour inoculation, draining LNs were analysed for OVA-specific CD8^+^ T cells using OVA-Ip tetramer (**d**). OVA-Ip-specific CD8^+^ T-cell response was also evaluated by the IFN-γ ELISPOT assay (**e**). Data shown are mean±s.e.m. with *n*=4–5 per group. (**f**) CD62L^lo^ effector OT-II cells primed in young or aged IL-6^+/+^ or IL-6^−/−^ mice were sorted as in Fig. 4a and were co-cultured with CFSE-labelled CD8^+^ T cells in the presence or absence of anti-IL-10 Ab. After 3 days, CFSE profile and IFN-γ production in CD8^+^ T cells were determined using a flow cytometer. Representative data from two independent experiments are shown. *P<0.05, **P<0.01, ***P<0.001, analysis of variance followed by Tukey's *post hoc* test.

To further clarify the inhibitory effect of IL-6 on Th1 differentiation, young CD4^+^ T cells were stimulated with anti-CD3/CD28 Abs in the presence or absence of IL-6 *in vitro*. In the presence of IL-12, stimulated T cells upregulated T-bet expression (Fig. 8a). Simultaneous IL-6 stimulation did not change the levels of either T-bet or GATA-3, but upregulated c-Maf expression even in the Th1-skewed condition. To directly assess the requirement of c-Maf for IL-6-mediated Th1 suppression, we utilized T cells with a homozygous mutant in c-Maf that lacks DNA-binding activity to its target genes^27^. c-Maf activity is dispensable for the development and peripheral homeostasis of T cells (Supplementary Fig. 6a,b). Proliferation of c-Maf-mutant naive CD4^+^ T cells in response to antigenic stimulation was also comparable to that of WT cells (Supplementary Fig. 6c). As shown in Fig. 8b, stimulation of WT cells with IL-6, which mimicked the aged environment, attenuated the development of IFN-γ-producing cells *in vitro*. Under these conditions, there was only subtle inhibition of Th1 differentiation by IL-6 in c-Maf-mutant T cells, suggesting the importance of c-Maf in IL-6-mediated Th1 suppression.

c-Maf is a critical transcription factor to induce IL-4 and IL-21 production^25^,^28^. Indeed, IL-6-stimulated CD4^+^ T cells from both young C57BL/6 and Balb/c mice produced significantly higher amounts of IL-4 immediately after primary T cell receptor (TCR) stimulation, and this was diminished in c-Maf-mutant T cells (Supplementary Fig. 7a,b; Fig. 8c). IL-6 stimulation also induced IL-21 and IL-10 production in a c-Maf-dependent manner (Fig. 8b,c; Supplementary Fig. 7). The c-Maf-dependent-altered profile of cytokine expression raised the possibility that these cytokines contributed to the IL-6-mediated suppression of Th1 differentiation. To test this possibility, Th1 differentiation was assessed in IL-6-stimulated T cells when these cytokine activities were neutralized. Neutralization of IL-4 activity significantly improved Th1 differentiation (Fig. 8d; Supplementary Fig. 7d). Blocking of IL-21 activity also mitigated IL-6-mediated Th1 suppression to a lesser extent, whereas exogenous IL-21 attenuated Th1 differentiation in the absence of IL-6 stimulation (Fig. 8e). Of note, these *in vitro* results were confirmed by the fact that neutralization of both IL-4 and IL-21 significantly restored the Th1 differentiation in aged mice (Fig. 9a), while IL-10 blockade did not improve the attenuated Th1 differentiation (Fig. 9b). Collectively, these results suggest that IL-6-induced Th1 suppression was mediated through a c-Maf/IL-4/IL-21 axis in the aged environment.

### IL-6-altered cytokine milieu modulates CD8^+^ T-cell responses

Consistent with IL-6-dependent IL-10 production in OT-II cells (Fig. 4c), increased level of IL-10 in the serum was observed in aged mice after tumour inoculation (Fig. 9c), while this increase was not detected in aged mice with IL-6 blockade. Thus, to explore the effect of IL-10, in addition to IL-4 and IL-21, on induction of antitumour CD8^+^ T-cell responses through CD4 helper activity, the number and function of CD8^+^ T cells were assessed in aged mice treated with blocking Abs for these cytokines. In line with the results of Th1 differentiation (Fig. 9a), combined treatment of anti-IL-4 and anti-IL-21 Abs significantly reversed the impaired induction and function of tumour-specific CD8^+^ T cells in response to tumour inoculation in aged mice. IL-10 blockade also improved the CD8^+^ T-cell responses in aged mice although Th1 differentiation was not affected by IL-10 blockade, suggesting that in contrast to IL-4/IL-21, IL-6-mediated IL-10 production by CD4^+^ T cells primed in the aged environment modulated CD8^+^ T-cell response independently of Th1 differentiation.

To confirm the direct inhibitory role of IL-6-sensitized CD4^+^ T cells, we performed the co-culture of CD8^+^ T cells with effector OT-II cells primed in young or aged IL-6^+/+^ or IL-6^−/−^ mice (Fig. 9f). Co-culture with CD4^+^ T cells primed in aged IL-6^+/+^ mice markedly suppressed the expansion and IFN-γ production of CD8^+^ T cells, but these responses were substantially induced when co-cultured with effector CD4^+^ T cells isolated from aged IL-6^−/−^ mice, or with CD4^+^ T cells from aged IL-6^+/+^ mice in the presence of anti-IL-10 Ab *in vitro*. These findings suggest that excessive production of IL-4, IL-21 and IL-10 induced by IL-6 in aged mice have negative impacts on antitumour responses of CD8^+^ T cells through (1) attenuating Th1 differentiation and their helper activity and (2) a direct inhibitory effect on the expansion and function of CD8^+^ T cells.

## Discussion

Considering the broad effects of ageing on CD4^+^ T cells, one of principal causes for the failure to mount the antitumour immune responses is the quantitative declines in number and TCR repertories of peripheral T cells due to thymic involution in old age^12^,^13^,^29^. A second risk factor is thought to be impairment of antigen-induced T-cell expansion, which is regulated by both intrinsic mechanisms in aged CD4^+^ T cells^11^,^12^ and extrinsic factor(s) in the aged environment (Fig. 3)^14^. The inherent proliferative defect in aged CD4^+^ T cells is largely due to reduced IL-2 production^11^, whereas the molecular mechanism(s) by which the aged environment depresses young CD4^+^ T-cell expansion have not yet been defined; therefore further analyses will be required.

Given that the balance between Th1 and Th2 cytokines is important for antitumour responses^5^,^7^, particular attention has been devoted to evaluating the age-related decline in IFN-γ production. Although a shift from Th1 towards Th2 responses in elderly subjects has been suggested^8^,^9^, some reports are contradictory^30^. This discrepancy may be due to the experimental materials, in which the effector CD4^+^ T cells primed at old age were not distinguished from the memory T cells that had been committed at young age. To rule out such possibilities, we established a model system that enabled us to assess the differentiation from naive to effector CD4^+^ T cells in the aged environment. Using this system, we found that the excessive increase in IL-6 induced the dysregulation of Th1 differentiation, which was suggested to a third cause of impaired antitumour immunity in aged animals.

Young mice rejected the OVA-expressing tumour even when IFN-γ-deficient OT-II was transferred, because IFN-γ deficiency in the transferred OT-II cells could be counterbalanced by abundant IFN-γ-producing endogenous WT CD4^+^ T cells in young mice, which might be sufficient to mount antitumour immunity. In this regard, in aged mice, the effect of IFN-γ deficiency in the transferred OT-II cells appeared to be underscored by significantly decreased proportion of endogenous CD4^+^ T cells^13^,^29^. Taken together, not only complementation with tumour-specific young CD4^+^ T cells, but IL-6 blockade that augmented IFN-γ production was also necessary for improvement of antitumour responses in aged mice.

IL-6-mediated Th1 suppression may be involved in a recent finding that the level of IL-6 is correlated with clinical outcome in melanoma patients receiving immunotherapy^31^. Therefore, the effect of IL-6 on endogenous or transferred T cells should be taken into account in aged individuals therapeutically infused with tumour-targeting T cells^32^,^33^. Age-related chronic inflammation accompanied with cancer, smoking, subclinical disorder (atherosclerosis, sarcopenia and cognitive decline) and obesity seem to contribute to systemic increase of IL-6/sIL-6R in an elderly population^15^,^17^. Overall, we suggest that a rational design of cancer immunotherapy mediated by CD4^+^ T cells in the elderly can be administered concurrently with temporal neutralization of IL-6 activity to restore the defective Th1 development without adversely affecting their antitumour activities. Theoretically, depletion of cells producing IL-6 or sIL-6R might represent viable options to ameliorate their activities. However, under inflammatory conditions, diverse types of cells such as aged adipose tissue^34^, MDSC^26^,^35^, macrophages^36^, nonhematopoietic endothelial stromal cells and/or cancer-associated fibroblasts^37^ can serve as the sources of IL-6 in tumour-bearing mice. For this reason, it has to be considered that the production of IL-6 and sIL-6R are not confined to one cell type and thus it is difficult to target their cellular sources that contribute to systemic control of Th1 suppression.

At present, IL-6 blockade using humanized anti-IL-6 Ab^19^, anti-IL-6R Ab tocilizumab or curcumin, a potent inhibitor of IL-6 production^35^,is a promising clinical application for improvement of the currently inefficient antitumour vaccination in aged individuals, and for controlling the age-dependent pro-inflammatory status, called ‘inflammaging', responsible for tissue damage or systemic toxicities in aged animals^16^,^17^.

The detailed molecular mechanism(s) underlying the defect in Th1 development are still unclear, and several scenarios are worth considering. (1) It was intriguing that IL-4/IL-21 neutralization substantially relieved the suppressive effect of IL-6 on Th1 differentiation. This was supported by the finding that IL-6-mediated impairment of Th1 development was substantially restored by a loss of function in c-Maf. Diminished IL-4/IL-21 production in c-Maf-mutant T cells likely allowed them to differentiate into Th1 cells, since IL-4 inhibits IFN-γ production and stimulates the production of other Th2 cytokines^25^. Furthermore, in addition to previous *in vitro* study^38^, we have first demonstrated the IL-6-mediated regulation of c-Maf expression under *in vivo* situation of aged mice (Fig. 5). On the basis of these findings, immunotherapy for aged individuals aimed at cancer regression may be accomplished by overcoming or redirecting Th2-prone responses. However, IL-4/IL-21 blockade did not completely rescue the impaired Th1 differentiation in aged mice, suggesting an additional requirement for optimal Th1 development.

(2) In addition to the IL-4/IL-21-mediated Th1 suppression, direct engagement of c-Maf in repressing *ifng* transcription is also likely. This possibility is supported by a previous report that forced expression of c-Maf in Th1 cells inhibited IFN-γ production via an IL-4-independent mechanism^39^. Furthermore, in addition to increased c-Maf expression, expression of T-bet and GATA3 were downregulated in donor OT-II cells primed in aged mice, implying that mutually balanced expression of several transcription factors within T cells contributes to attenuated Th1 differentiation in the aged environment. (3) Moreover, as an alternative mechanism of IL-6-mediated Th1 suppression, impaired Signal Transducer and Activator of Transcription 1-dependent IFN-γ receptor signalling via IL-6-induced expression of Suppressor of Cytokine Signalling protein has been proposed^40^. These possible mechanisms should be investigated in greater detail.

Our finding provides the notion that less susceptibility to the antitumour vaccination in aged individuals should be carefully considered in terms of the complex effects of ageing on other components that interact with CD4^+^ T cells. In addition to the quantitative declines in aged CD8^+^ T cells, their qualitative defects have been also reported^41^. However, there are other conflicting data suggesting that intrinsic function of CD8^+^ T cells is preserved even in later life^42^,^43^. These seeming discrepancies can be explained in part by the defective ability of CD4^+^ T cells to help cognate CD8^+^ T cells in old age. Indeed, we found that CD4^+^ T cells primed in aged mice were ineffective in supporting cognate CD8^+^ T-cell responses, resulting in poor control of tumour progression in aged mice. Moreover, their helper activity for CD8^+^ T cells was mediated through IFN-γ, which was consistent with previous reports showing that CD4^+^ T-cell-derived IFN-γ enhanced the function and recruitment of CD8^+^ T cells into draining LNs following viral or bacterial infection^44^,^45^. Importantly, when Th1 differentiation was restored by the blockade of IL-6 or subsequent IL-4/IL-21 activities, induction of tumour-specific aged CD8^+^ T cells was enhanced as well, implying minimum intrinsic functional defects in aged CD8^+^ T cells.

It is noteworthy that IL-21 is known to have the ability to potentiate the expansion and function of CD8^+^ T cells in a synergistic context with IL-15 in young mice^46^, which is apparently opposed to our findings. One likely cause for this contradiction may be the reduced availability of IL-15 presentation in the aged environment, because the stimulatory effect of IL-21 is not exerted on CD8^+^ T cells in the absence of IL-15 (ref. 46). Indeed, previous studies reported that the levels of IL-15 and IL-15Rα decline with ageing^47^,^48^. These reports appear to be consistent with our hypothesis that insufficient cooperative action of IL-15 is responsible for the decreased stimulatory function of IL-21. However, it remains a matter of debate as to whether ageing alters levels of IL-15, because in contrast to these reports, a recent study demonstrated that IL-15 expression in splenic stromal cells was increased with ageing^49^.

Interestingly, our *in vitro* experiments demonstrated that IL-4/IL-21 blockade downregulated IL-10 production from IL-6-sensitized effector CD4^+^ T cells (Supplementary Fig. 7c), suggesting that IL-21 stimulated IL-10 production in CD4^+^ T cells, which in turn, constrained the optimal response of CD8^+^ T cells in an IL-10-dependent manner. Taken together, in addition to the negative impact on Th1 differentiation, this cytokine circuit could be another mechanistic feature of IL-6 in converting the function of IL-21 from immunostimulatory to tolerogenic for tumour outgrowth in aged animals. On the basis of these ideas, strategies that bypass the requirement of CD4^+^ T-cell-mediated help and compensate for apparently ‘helpless CD8^+^ T cells', such as treatment with agonistic anti-CD40L Ab (plus TNF-α blockade)^16^, or anti-PD-1 Ab^50^ may be the potential approaches to restore the ineffective cancer immunosurveillance in aged animals without distraction of defective CD4^+^ T cells.

## Methods

### Mice

Two-month-old young male C57BL/6 mice were purchased from Nihon Clea, and were maintained until they reached 18–24 months of age, which were utilized as aged mice. Two-month-old Balb/c mice (Nihon Clea) were also utilized as a source of naive CD4^+^ T cells. OVA-specific OT-II TCR-transgenic mice were crossed with B6.SJL-PtprcaPep3b/BoyJ (The Jackson Laboratory) or with IFN-γ-deficient mice^51^ to establish CD45.1^+^OT-II mice or IFN-γ-deficient OT-II mice, respectively. C3H/HeH F1 background Maf^*Ofl*^ mice bearing a mutant c-Maf that lacks the ability to bind to its target genes^27^ were provided from the Medical Research Council. All mice including IL-6-deficient mice were housed at the Center for Animal Resources and Development, Kumamoto University under specific pathogen-free conditions. All the experimental procedures were performed in accordance with the guidelines of the Institutional Animal Committee of Kumamoto University.

### Tumour inoculation and Ab treatment *in vivo*

An MCA205 fibrosarcoma cell line expressing OVA protein was previously established^21^. RMA cells, a Rauscher MuLV-induced lymphoma^21^ was kindly provided by Dr Akira Shibuya (University of Tsukuba, Japan). Mice were inoculated subcutaneously with 8 × 10^5^ MCA-OVA or 1 × 10^5^ RMA, or were intravenously injected with 1 × 10^5^ of the OVA/firefly luciferase-expressing melanoma cell line, MO4-Luc^26^,^52^, in the pulmonary metastatic model. Tumour size is expressed as a tumour index, the square root of (length × width), as described previously^53^. To measure MO4 metastasis in the lung, luminescence images were analysed using NightOWL II (Berthold Technologies)^26^. Two hundred μg of anti-IL-6R Ab, 15A7; anti-IL-6 Ab, MP5–20F3 (BioXCell); or control rat IgG Ab (Millipore) was injected 1 day before and after immunization. Daily intraperitoneal injections of 200 μg of anti-IL-4 Ab (BioXCell) and 100 μg of anti-IL-21 Ab (R&D Systems) were performed for 2 days after T-cell transfer. Two hundred μg of anti-IL-10 Ab (JES5-2A5; BioXCell) was injected every other day after tumour inoculation. For *in vivo* cell depletion, mice were injected with 200 μg of anti-CD4 Ab (GK1.5), anti-CD8 Ab (2.43) or anti-Gr-1 Ab (R56–86; BioXCell) 4–5 days before T-cell transfer, immunization or tumour inoculation.

### Adoptive transfer and immunization

Naive CD4^+^ T cells (1 × 10^6^) isolated from OT-II TCR-transgenic mice using the naive CD4^+^ T-cell isolation kit (Miltenyi Biotec) were injected intravenously into mice. Eight hours after the T-cell transfer, mice were immunized by an intravenous injection of 4 × 10^5^ young bone marrow-derived DCs^26^ pulsed with peptide ISQAVHAAHAEINEAGR, which is recognized by I-A^b^-restricted OT-II cells, or peptide SIINFEKL, which is recognized by H2-K^b^-restricted OVA-specific CD8^+^ T cells (referred to as OVA-IIp or OVA-Ip, respectively). Alternatively, OVA-IIp emulsified in IFA was subcutaneously injected. Transfer of DCs pulsed with MuLV Env-gp70 H13.3 peptide SLTPRCNTAWNRL (EnvH13.3) was also performed for the vaccination against RMA tumour progression^21^. Labelling with CFSE (Dojindo) was performed according to the manufacturers' instructions.

### Flow cytometric analysis

Tumour-infiltrated lymphocytes were prepared from tumours by enzymatic digestion with 2.5 mg ml^−1^ collagenase D (Roche) and 0.1 mg ml^−1^ DNase I (Sigma) for 30 min at 37 °C. Cell suspensions from blood, spleen, LNs and tumour were stained with the following antibodies for flow cytometric analysis: anti-CD11b (1:400), anti-Vβ5 (1:200), anti-CD8 (1:200), anti-CD4 (1:400), anti-CD27 (1:100), anti-CD25 (1:400), anti-CD69 (1:400) Abs and PerCP-streptavidin (1:400) were purchased from BD Biosciences. Anti-Ly6C, (Biolegend), anti-Gr-1 (1:100), anti-CD45.1 (1:200), anti-Ly6G (1:100), anti-CXCR3 (1:100), anti-CD127 (1:100), anti-PD-1 (1:100), anti-PD-L1 (1:200), anti-ICOS (1:200) and anti-OX40 (1:100) Abs were purchased from eBioscience. The H-2K^b^/SIINFEKL-OVA tetramer-PE was purchased from MBL (1:50). For cytokine staining, CD4^+^ T cells that were isolated from mice using anti-CD4 beads or from *in vitro* cultures were re-stimulated with OVA-IIp-pulsed DCs or Phorbol-12-Myristate-13-acetate/ionomycin, and then were stained with anti-IL-2 (1:400), anti-IL-17A (1:100), anti-IL-10 (1:100), anti-IFN-γ (2:100), (eBioscience) or anti- TNF-α Abs (1:400; Biolegend)^54^. Intracellular staining of IL-21 was performed by using IL-21R-Fc chimeric protein (R&D Systems) and fluorescein-conjugated anti-human IgG (Jackson ImmunoResearch)^28^. Staining with anti-T-bet (1:200), anti-GATA-3(1:50), anti-RORγt (1:50), anti-Foxp3 (1:50) or anti-c-Maf Abs (1:100) were performed using the Transcription factor buffer set (BD Biosciences). Immunofluorescence images were analysed using the FACSVerse or FACSCalibur (Becton Dickinson). Data were analysed using FlowJo software (Treestar).

### Real-time quantitative PCR

Total RNA from isolated CD4^+^ T cells was extracted using the RNeasy Plus Mini Kit (Qiagen), and then was reverse-transcribed with the ReverTra Ace (TOYOBO). Real-time quantitative PCR was performed in triplicate on the ViiA7 Real-Time PCR System with TaqMan probes and Master Mix reagents (Applied Biosystems). Each gene expression was normalized to the expression of mouse *Gapdh* on the basis of the comparative 2^[−ΔΔCT]^ method. TaqMan probes were as follows: *c-maf* (Mm02581355_s1), *Tbx21* (Mm00450960_m1), *Gata3* (Mm00484683_m1), *Rorc* (Mm01261022_m1), *Foxp3* (Mm00475162_m1), *Il10* (Mm01288386_m1), *Bcl6* (Mm00477633_m1), *Il17a* (Mm00439618_m1), *Il4* (Mm00445259_m1), *Ifng* (Mm01168134_m1), *Il21* (Mm00517640_m1) and *Gapdh* (Mm99999915_g1).

### Homeostatic expansion

To analyze homeostatic division, young and aged mice were lethally irradiated with 9.5 Gy in two doses of 4.75 Gy each, with a 6-h interval. These irradiated mice were then intravenously transferred with CFSE-labelled young OT-II cells (1 × 10^6^).

### Proliferation assay

To assess the primary T-cell expansion, naive CD4^+^ T cells (2.5 × 10^4^ per well) were isolated from homozygous Maf^*Ofl/Ofl*^ mice or littermate control mice, and then stimulated with plate-coated anti-CD3 Ab (2 μg ml^−1^) and anti-CD28 Ab (5 μg ml^−1^). After 48 h of *in vitro* culture, T cells were pulsed with [^3^H]-thymidine, and the incorporation of radiolabel was determined by counting radioactivity 18 h later.

### Cytokine measurement

CD62L^lo^ effector CD4^+^ T cells were isolated using the CD4^+^ T-cell isolation kit, followed by negative selection with the CD62L MACS beads (Miltenyi Biotec) from mice that were transferred OT-II cells and immunized with OVA-IIp-pulsed DCs, and then the T cells (3 × 10^4^) were re-stimulated with OVA-IIp-pulsed DCs for 36 h *ex vivo*. Cytokine levels in culture supernatants were measured using the custom Bio-Plex Pro assay kit and suspension array system (Bio-Rad). IL-6 and soluble IL-6R (sIL-6R) levels in serum, or IL-4 and IL-21 levels in supernatants were determined using the enzyme-linked immunosorbent assay (R&D systems).

For the mouse enzyme-linked immunospot (ELISPOT) assay (BD Biosciences), draining LN cells (1 × 10^5^ cells per well) and bone marrow-derived DCs (3 × 10^4^) pulsed with OVA-Ip, OVA-IIp, D^b^-binding MuLV GagL peptide (LCCLCLTVFL) or K^b^-binding Env peptide (SSWDFITV)^21^ were added to wells of ELISPOT plates in triplicate and incubated for 12 h. IFN-γ spots were visualized with biotin-conjugated IFN-γ detection Ab, avidin-horseradish peroxidase and 3-amino-9-ethylcarbazole substrate reagent (BD Biosciences). ELISPOT plates were analysed using Eli photo (Minerva Tech, Tokyo, Japan).

### *In vitro* Th1 differentiation

Naive T cells from young mice were stimulated with plate-bound anti-CD3 and anti-CD28 Abs (both eBioscience) in the presence of IL-12 (8 ng ml^−1^; Wako) at 2.5 × 10^5^ cells ml^−1^ for 5 days. IL-6 (10 ng ml^−1^; Peprotech), IL-21 (20 ng ml^−1^; Wako), anti-IL-4 Ab (10 μg ml^−1^) and/or anti-IL-21 Ab (10 μg ml^−1^; R&D Systems) was added to the culture.

### CD8^+^ T-cell suppression assay

CD62L^lo^ effector CD4^+^ T cells primed in young or aged mice were isolated using CD4 isolation kit, followed by depletion of non-CD62L^hi^ cells with CD62L MACS beads (Miltenyi Biotec). Sorted CD4^+^ T cells were co-cultured with CFSE-labelled naive CD8^+^ T cells in anti-CD3 and anti-CD28 Abs-coated plates at CD4^+^/CD8^+^ T-cell ratios of 4:1. After 3 days, proliferation and IFN-γ production in CD8^+^ T cells were determined by CFSE dilution and intracellular IFN-γ staining, respectively. IL-10 activity was neutralized by the addition of anti-IL-10 Ab (10 μg ml^−1^).

### Statistical analysis

All statistical analyses were performed using Prism 4.0 software (GraphPad). Multiple group comparisons were performed by one-way analysis of variance followed by Tukey's *post hoc* tests. Data were also analysed using an unpaired Student's *t*-test when comparing two experimental groups. *P* values less than 0.05 were considered significant.

## Author contributions

H.T. conceptualized the research, designed and performed the experiments, analysed the data, and wrote the manuscript. S.S. analysed the data and edited the manuscript. K.M. performed research. S.L.S. conceptualized the research, analysed the data and wrote the manuscript. H.T. and Y.N. directed the study and edited the manuscript.

## Additional information

**How to cite this article:** Tsukamoto, H. *et al.* IL-6-mediated environmental conditioning of defective Th1 differentiation dampens antitumour immune responses in old age. *Nat. Commun.* 6:6702 doi: 10.1038/ncomms7702 (2015).

## Supplementary Material

Supplementary Information Supplementary Figures 1-7
