# Supplementary material for: IL-6-mediated environmental conditioning of defective Th1 differentiation dampens antitumour immune responses in old age
Source: Nat Commun. 2015 Apr 7;6:6702. doi: 10.1038/ncomms7702 (PMC4396369; doi:10.1038/ncomms7702)
Supplement: Supplementary Information — Supplementary Figures 1-7 [file ncomms7702-s1.pdf]

Supplementary Figure 1

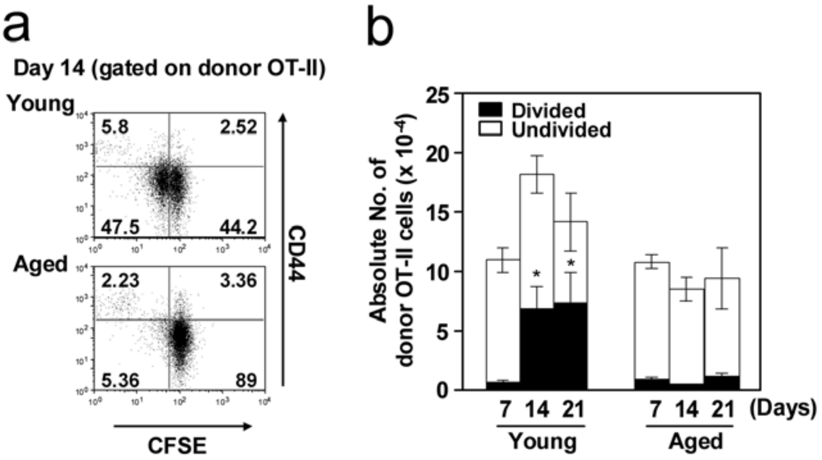

Supplementary Figure 1. Homeostatic expansion of young OT-II cells in young and aged environments. CFSE-labeled young OT-II cells were transferred into irradiated young or aged hosts. The indicated days after transfer, donor OT-II cells were harvested from spleen and LNs. CFSE intensity and CD44 expression (**a**) and the absolute number (**b**) of donor OT-II cells are shown. "Divided" and "Undivided" represent $CFSE^{lo}$ and $CFSE^{hi}$ population indicated in **a**, respectively. The data are representative of three independent experiments (mean ± SEM with n = 4). Asterisks indicate the significance of the difference between young and aged group; * $p < 0.05$ (one-way ANOVA followed by Tukey's post hoc tests).

Supplementary Figure 2

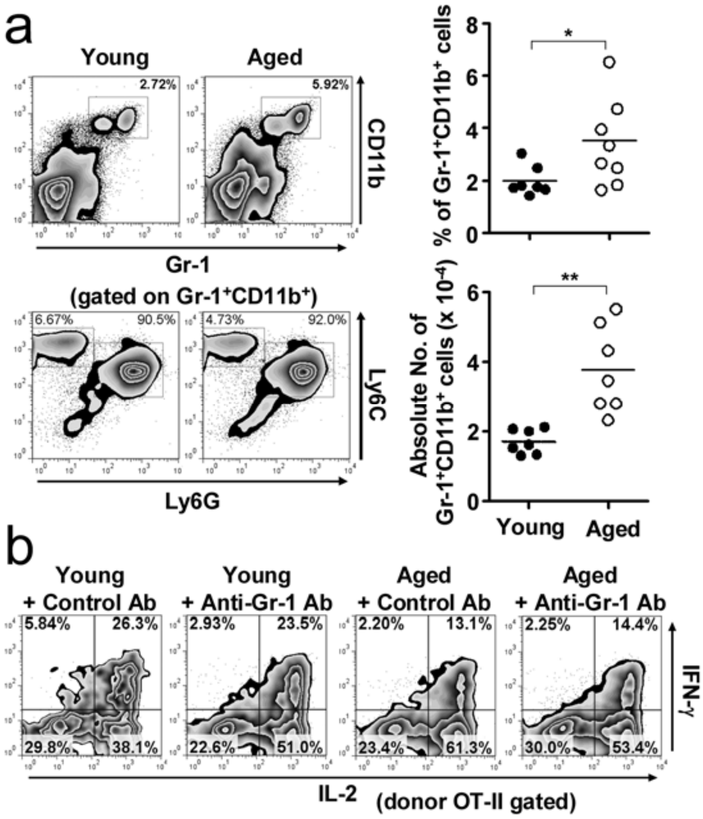

Supplementary Figure 2. Gr-1$^+$CD11b$^+$ cells increase in aged mice. (**a**) Spleen and lymph node cells were harvested from young or aged mice, and Gr-1$^+$CD11b$^+$ MDSC were analyzed. Representative plots of Gr-1 and CD11b expression (upper left) and Ly6C and Ly6G expression in the gated population (lower left), and total number of Gr-1$^+$CD11b$^+$MDSC (right) are shown (mean with n = 7-8). (**b**) Young naïve OT-II cells were primed *in vivo* as described in Figure 2. Mice were treated with control Ab or anti-Gr-1 Ab 4 days before immunization (n = 4/group). Seven days after immunization, donor OT-II cells were re-stimulated with PMA/ionomycin. Representative plots of IFN-γ and IL-2 production are shown. Representative data from three experiments are shown. Asterisks indicate the significance of the difference between young and aged group; * $p < 0.05$, ** $p < 0.01$ (unpaired Student's *t*-test).

Supplementary Figure 3

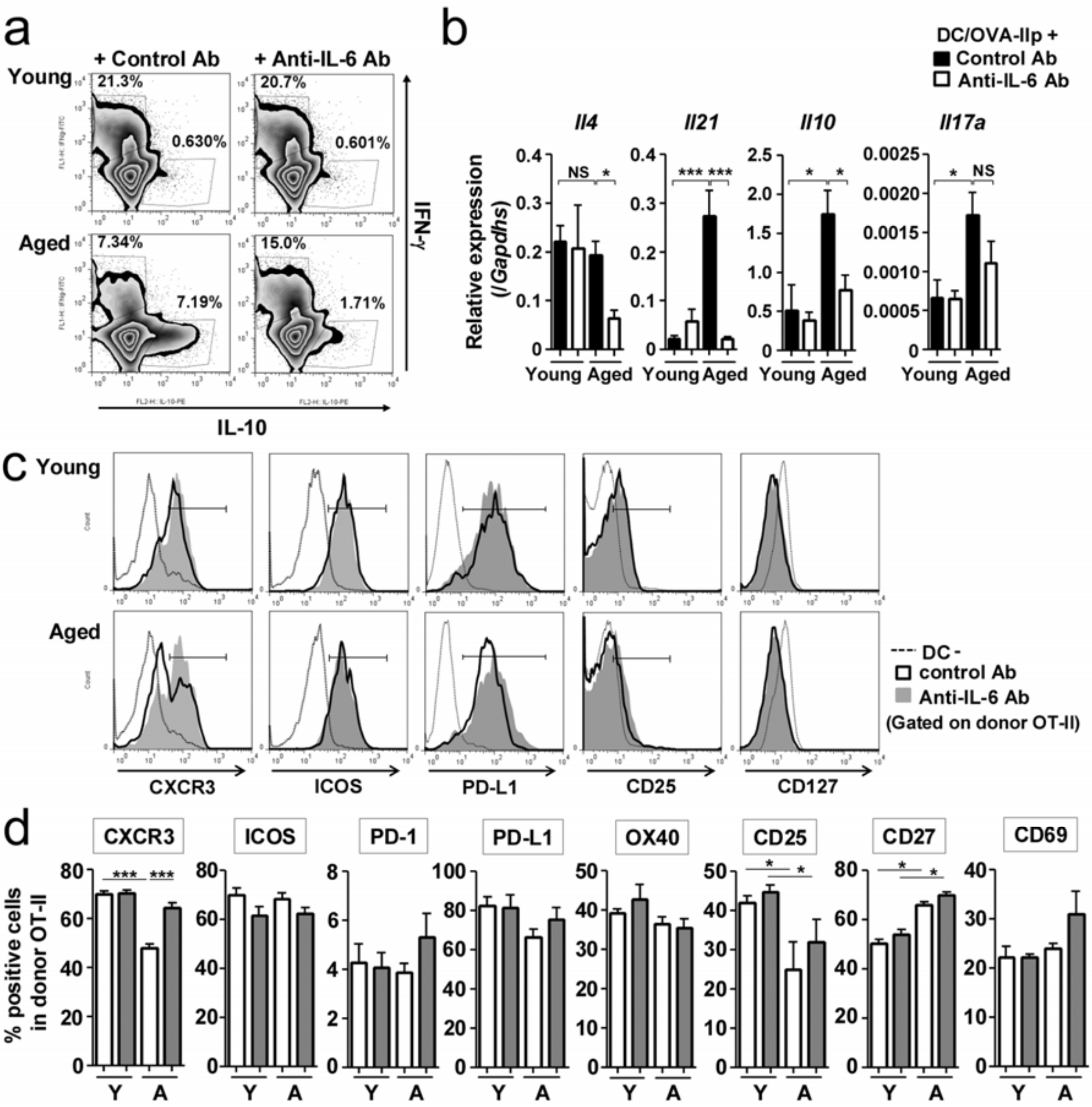

Supplementary Figure 3. Characterization of donor OT-II cells primed in young or aged mice. Young naïve OT-II cells were transferred into young or aged mice, and then were primed *in vivo* as described in Fig. 1d. Mice were treated with control Ab or anti-IL-6 Ab 1 day before immunization. (**a**) Five days after immunization, isolated $CD4^+$ T cells were re-stimulated with PMA/ionomycin. Representative plots of IFN-γ and IL-10 production in gated donor OT-II cells are shown. (**b**) Four days after immunization, isolated $CD4^+$ T cells were analysed for the mRNA expression of the indicated genes by real-time qPCR. Each gene expression was normalized to the expression of mouse *Gapdh.* (**c** and **d**) Four or five days after immunization, the expression of indicated cell surface molecules on donor OT-II cells were assessed by flow cytometry. Representative histograms (c) and the frequencies of positive cells (d) in donor OT-II cells are shown. Two or three independent experiments were performed, and the values shown are mean ± SEM with n = 4-5 /group. Asterisks indicate the significance of the difference between indicated groups; * $p < 0.05$, ** $p < 0.01$, *** $p < 0.001$ (one-way ANOVA followed by Tukey's post hoc tests).

Supplementary Figure 4

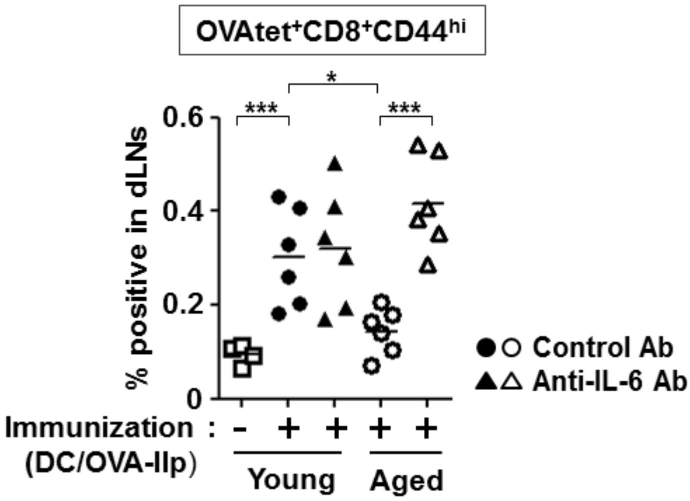

Supplementary Figure 4. Th1-dependent helper activity for tumour-specific CD8$^+$ T cells is diminished by IL-6 in aged mice. CD45.1$^+$ young naïve OT-II T cells were transferred into young or aged CD45.2$^+$ mice. The mice were then immunized by transfer of DC pulsed with or without OVA-IIp. Mice were treated with control or anti-IL-6 Ab on day -1 and day 1. Five days after immunization, MCA-OVA were inoculated. Another 6 days later, OVA-specific CD8$^+$ T cells in tumour draining LNs were analysed by flow cytometry. Representative data from three experiments are shown (mean with n = 4-6 /group). * $p < 0.05$, *** $p < 0.001$, ANOVA followed by Tukey's posttest.

Supplementary Figure 5

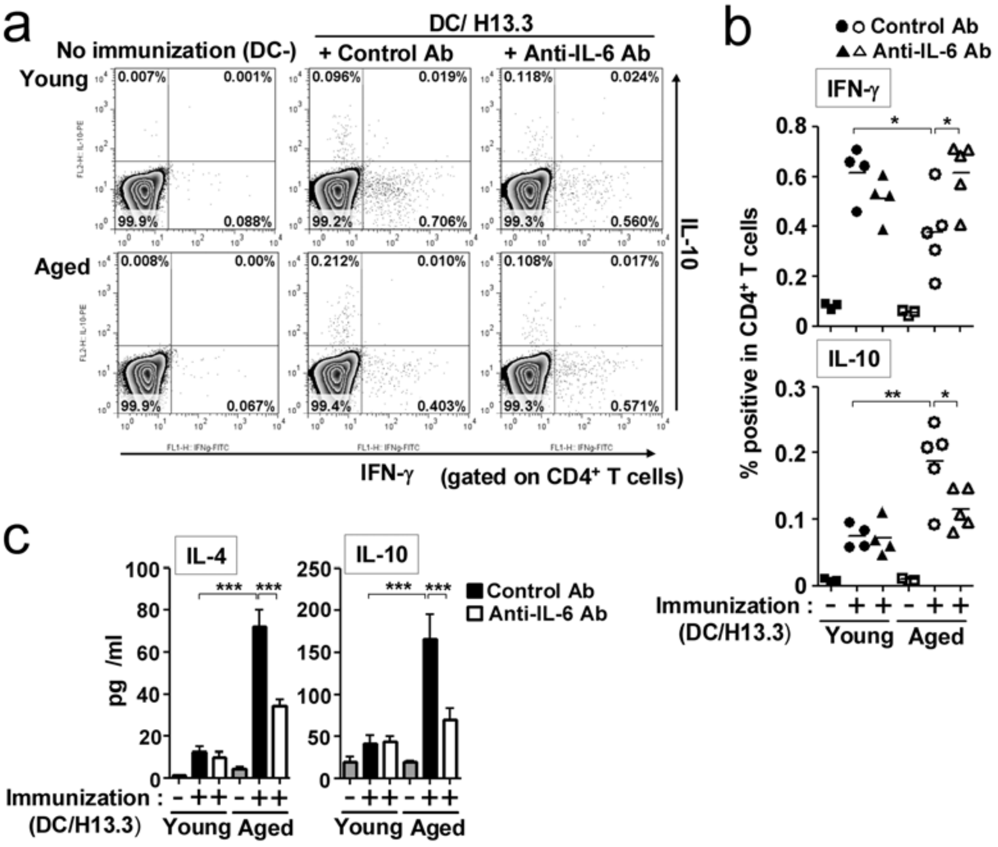

Supplementary Figure 5. IL-6 controls the reciprocal regulation of IFN-γ and IL-4/10 responses in tumor-associated antigen-specific CD4$^+$ T cells. (**a** and **b**) Young or aged mice were immunized by the transfer of DC pulsed MuLV EnvH13.3 peptide to prime cognate CD4$^+$ T cells. Anti-IL-6 Ab or control Ab was injected 1 day before and after immunization. Five days after immunization, RMA tumor cells were inoculated in the mice. Further 5 days later, draining LNs were harvested and re-stimulated with H13.3 peptide-pulsed DC. Representative dot plots of intracellular cytokine staining (a) and the frequencies (b) of IFN-γ- or IL-10-positive cells in gated CD4$^+$ T cells were shown. (**c**) Draining LN cells were stimulated with H13.3 peptide-pulsed DC for 36 h. The concentration of IL-4 or IL-10 in the culture supernatant was assessed by ELISA. The values are mean ± SEM with n = 4-5 mice/group. Data are representative from two independent experiments. Asterisks indicate the significance of the difference between indicated groups; * $p < 0.05$, ** $p < 0.01$, *** $p < 0.001$ (one-way ANOVA followed by Tukey's post hoc tests).

Supplementary Figure 6

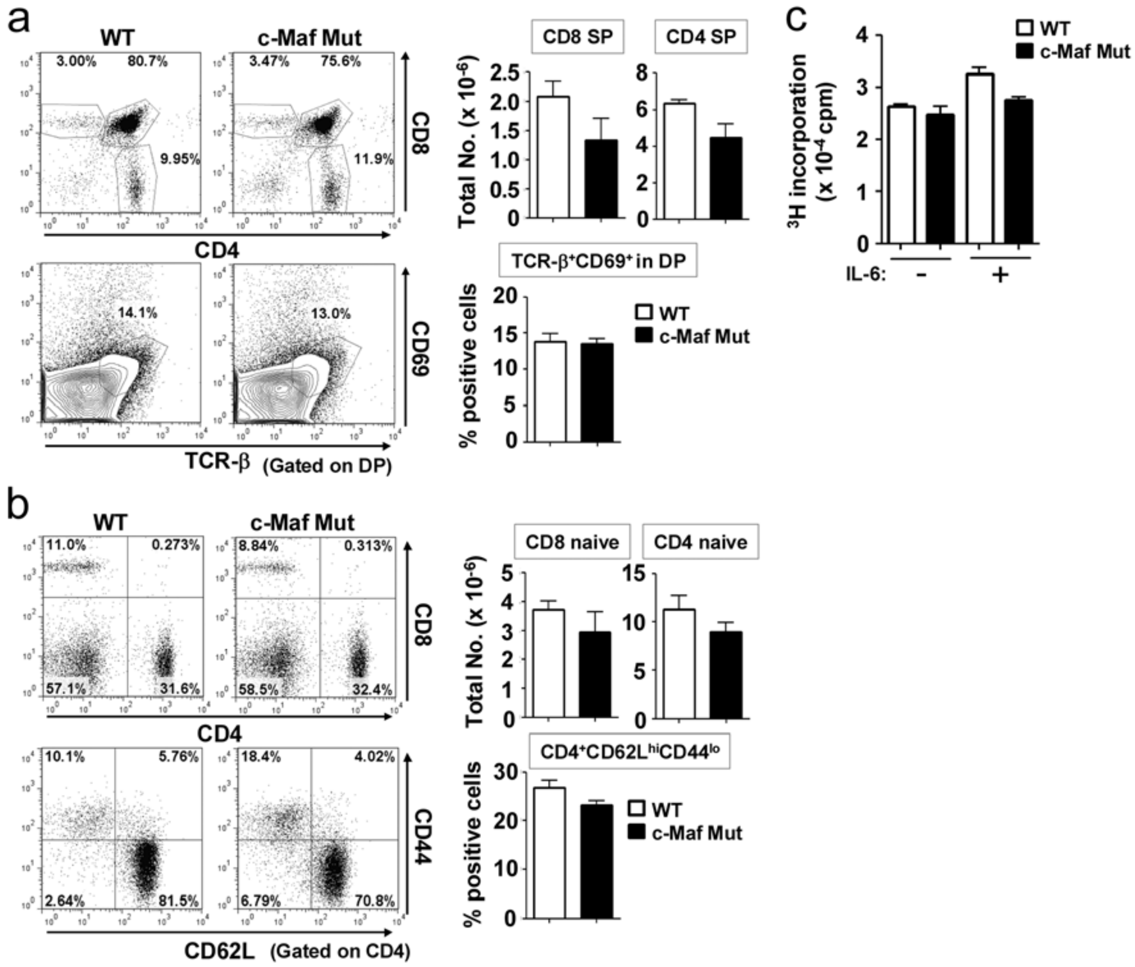

Supplementary Figure 6. Normal development and proportion of T cells in Maf$^{Ofl/Ofl}$ mutant mice. (**a**) Frequencies of CD4$^+$CD8$^-$, CD4$^-$CD8$^+$ single positive (SP), CD4$^+$CD8$^+$ double positive (DP) thymocytes in the thymus (upper left 2 panels), and TCR-β^{hi}CD69$^+$ cells in gated CD4$^+$CD8$^+$ DP thymocytes (lower left panels) of homozygous c-Maf-mutant mice (c-Maf Mut) or littermate control mice (WT) were determined by flow cytometry. Absolute numbers of CD4$^+$CD8$^-$ or CD4$^-$CD8$^+$ thymocytes are also shown (right). (**b**) Frequencies of CD4$^+$CD8$^-$, CD4$^-$CD8$^+$ T cells in the spleen and lymph nodes (upper left 2 panels), and peripheral naïve (CD44^{lo}CD62L^{hi}) CD4$^+$ T cells in the periphery are shown. Absolute numbers of CD44^{lo}CD4$^+$ or CD44^{lo}CD8$^+$ naïve T cells in the spleen and lymph nodes are also shown (right). The data are mean ± SEM with n = 3-4 mice/group. (**c**) Young naïve CD4$^+$ T cells were isolated from homozygous c-Maf-mutant mice (c-Maf Mut) or littermate control mice (WT), and then were stimulated with plate-coated anti-CD3 and anti-CD28 Abs, and then their proliferation were determined by [^{3}H] thymidine incorporation in triplicate assay. Data are one of two independent experiments and are mean ± SEM with n = 3-4 mice/group.

Supplementary Figure 7

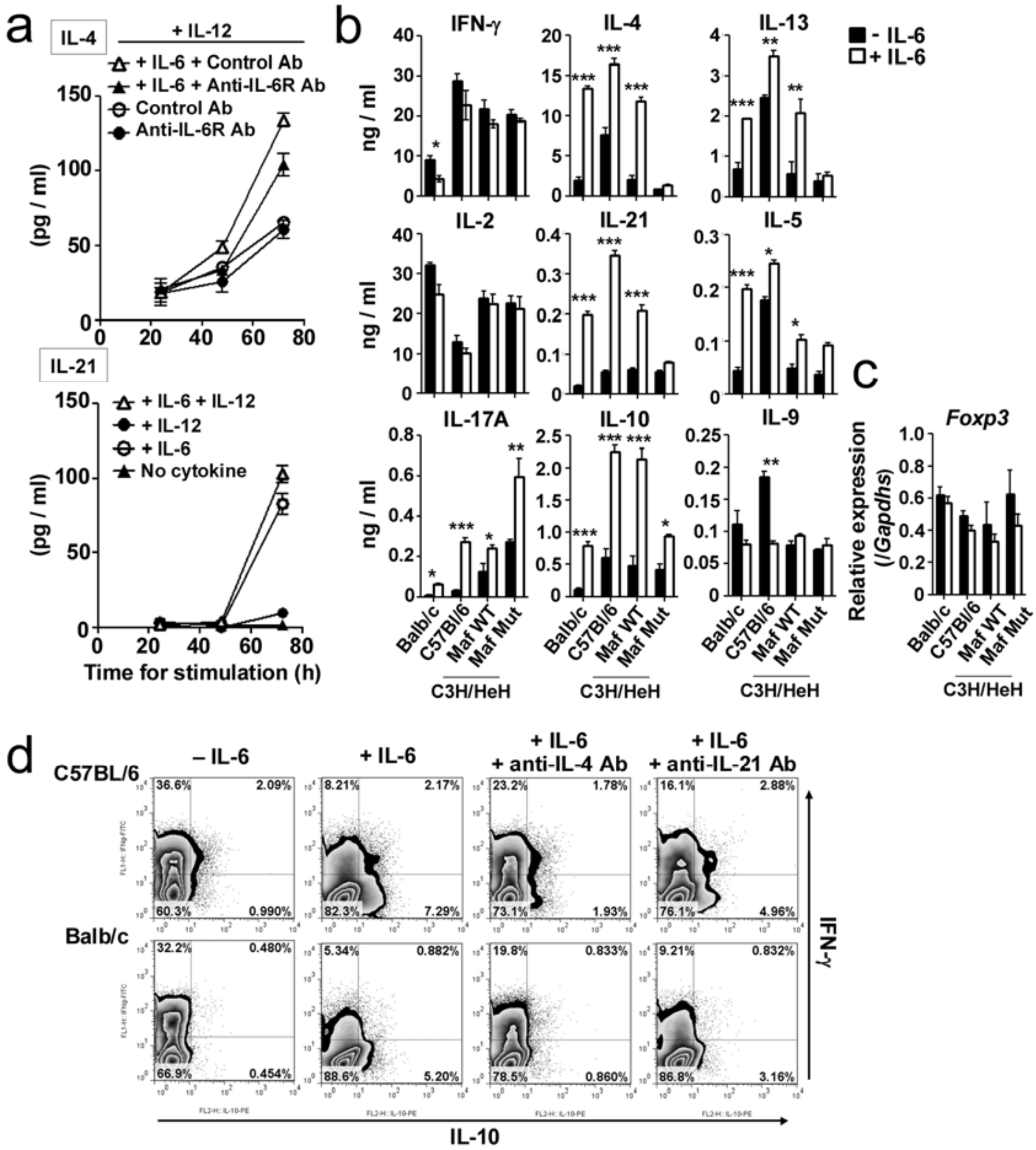

Supplementary Figure 7. IL-6 stimulation alters the early cytokine productions in activated CD4^{+} T cells through the c-Maf activity. (**a**) Young naïve OT-II cells were stimulated with anti-CD3 and anti-CD28 Abs *in vitro*. Indicated cytokines and Abs were also added at the initiation of culture. At the indicated time points after primary stimulation, the culture supernatants were collected, and then IL-4 and IL-21 concentrations were analysed by ELISA in triplicate assay. (**b** and **c**) Polyclonal naïve CD4^{+} T cells were isolated from young Balb/c, C57BL/6 or C3H/HeH (WT amd c-Maf$^{Ofl/Ofl}$ mutant) background mice, and then were stimulated in the presence or absence of exogenous IL-6 under Th1-skewed condition (plus IL-12). Sixty hours later, indicated cytokine concentrations in the culture supernatant were determined. mRNA was also isolated to examine the gene expression of *Foxp3* by qPCR (c). The values are mean ± SEM with n = 3 mice/group. Statistical significant differences between IL-6-sensitized and non-sensitized conditions in each strain are expressed as asterisks; * $p < 0.05$, ** $p < 0.01$, *** $p < 0.001$ (one-way ANOVA followed by Tukey's post hoc

tests). (**d**) Polyclonal naïve $CD4^+$ T cells were isolated from young Balb/c, or C57BL/6 background mice, and then were stimulated in the presence or absence of IL-6 and indicated Abs under Th1-skewed condition (plus IL-12). Five days later, T cells were re-stimulated and analyzed for the ability to produce IFN-γ and IL-10. All data shown are representative of 2 or 3 independent experiments.
